# Screening and diagnosis of liver disease in diabetes: a guide and evidence-based assessment

**DOI:** 10.1007/s00125-026-06816-0

**Published:** 2026-08-06

**Authors:** Sami F. Qadri

**Affiliations:** 1 Department of Medicine, University of Helsinki and Helsinki University Hospital, Helsinki, Finland; 2 Minerva Foundation Institute for Medical Research, Helsinki, Finland

**Keywords:** Bayesian analysis, Biomarkers, Cost-effectiveness, Diagnosis, Fatty liver, Fibrosis, Metabolic dysfunction-associated steatotic liver disease, Prevalence, Review, Screening, Type 2 diabetes

## Abstract

Most people with type 2 diabetes have metabolic dysfunction-associated steatotic liver disease (MASLD), yet fewer than 2% experience major adverse liver outcomes within 10 years. Because progression to end-stage liver disease is uncommon, identifying the small subset of individuals at highest risk remains a central clinical challenge. Liver fibrosis is the strongest prognostic determinant in MASLD, and a range of blood-based and imaging tests are used to assess the likelihood of advanced fibrosis (AF) in the clinic. Since 2023, major medical societies have recommended systematic screening for MASLD-related AF in individuals with type 2 diabetes, driven by reports suggesting a high prevalence of up to 20–40%. This review critically examines the evidence underlying these recommendations while providing a clinically oriented guide to diagnosing MASLD in type 2 diabetes. Re-analysis of data from prevalence studies suggests that the burden of AF in type 2 diabetes has been substantially overestimated and may be below 5–10%, which has profound implications for both the rationale for screening and its expected yield. Simulations and emerging real-world evidence suggest that current non-invasive tests may refer 30–50% of patients for costly second-line testing and 15% for specialist evaluation, while generating high false-positive rates and missing more than a third of all AF cases. Moreover, it remains uncertain whether diagnosing AF in type 2 diabetes currently leads to meaningful changes in management or outcomes. Existing cost-effectiveness analyses rely on assumptions about disease burden and therapeutic benefit that are not supported by available data. Together, these considerations call for a more rigorous, evidence-based approach to evaluating liver disease risk in type 2 diabetes and other populations.

**Graphical Abstract:**

**Figure:**
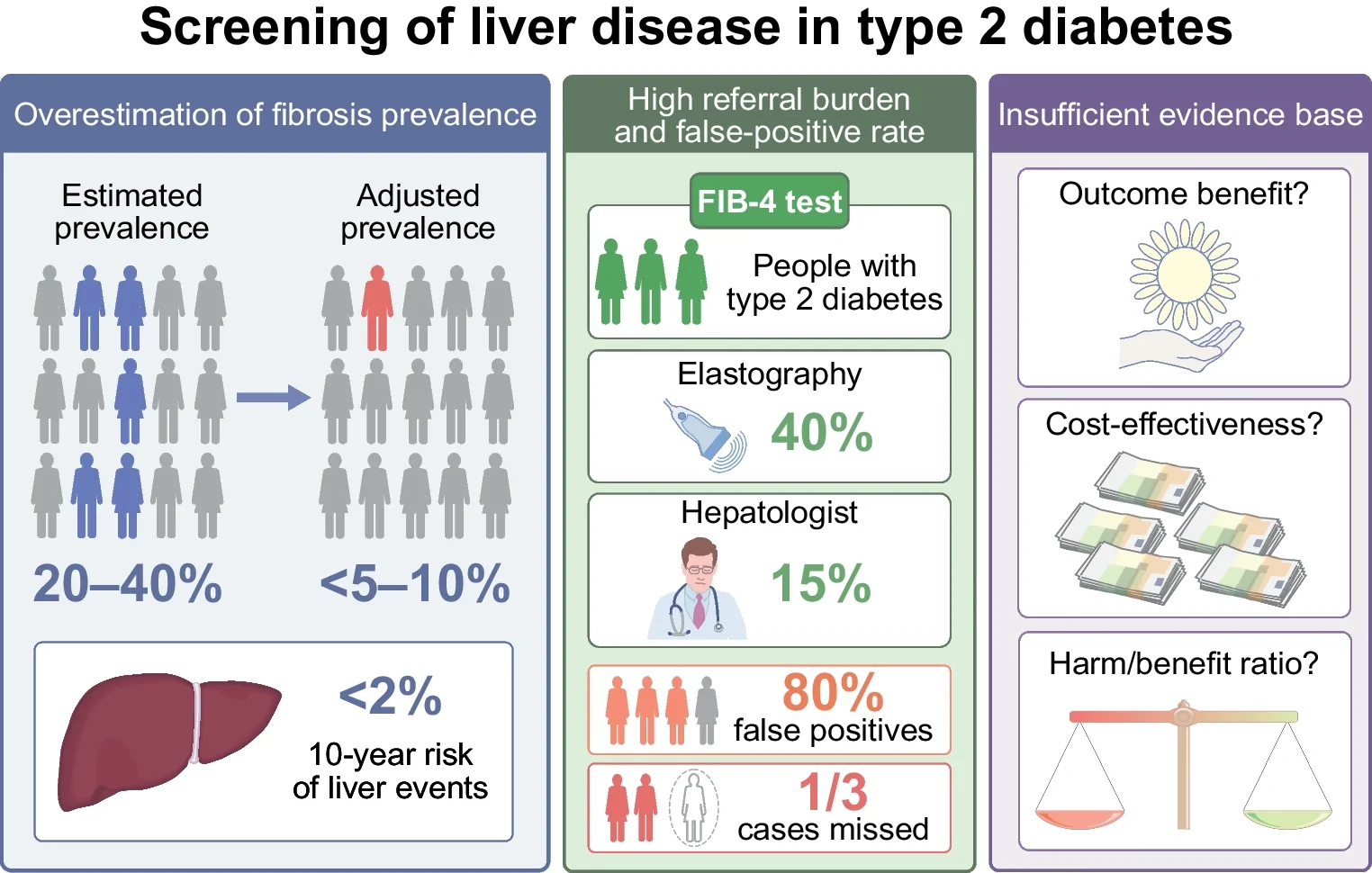

**Supplementary Information:**

The online version contains a slideset of the figures for download available at https://doi.org/10.1007/s00125-026-06816-0.

## Introduction

The central role of the liver in type 2 diabetes is reflected by the observation that 60–70% of individuals with type 2 diabetes have pathological hepatic fat accumulation [1, 2]. This condition, termed metabolic dysfunction-associated steatotic liver disease (MASLD), is the most prevalent chronic liver disorder worldwide and is now recognised as a major comorbidity of type 2 diabetes [3, 4]. Although MASLD can progress to end-stage liver disease, most individuals have isolated steatosis and follow an indolent course. Only a minority develop metabolic dysfunction-associated steatohepatitis (MASH) and fibrosis, and a small fraction ultimately progress to cirrhosis [5]. This heterogeneity creates a central clinical challenge: given the high population prevalence of MASLD, how can diagnostic and treatment efforts be targeted to those most likely to benefit?

Recognition that fibrosis, rather than steatosis, is the main prognostic determinant in MASLD has shifted attention towards identifying advanced fibrosis (AF), which is associated with a clinically significant 10 year risk (>5–10%) of major adverse liver-related outcomes (MALOs) [5]. Diagnosing AF may enable targeted use of intensive lifestyle, pharmacological and surgical interventions with proven benefit to ameliorate fibrosis [6–9], as well as prevention and earlier detection of cirrhosis-related complications. Since 2023, major professional societies have begun recommending systematic screening for MASLD-related AF in individuals with type 2 diabetes [4, 10, 11]. This shift was driven in part by reports suggesting that AF prevalence may be as high as 20–40%, with more rapid progression of MASH in this population.

This review provides a clinically oriented guide to evaluating MASLD in type 2 diabetes, including a focused appraisal of commonly used diagnostic tools and their limitations. It also critically examines both the rationale for AF screening and the feasibility of recommended screening strategies. A re-analysis of results from prevalence studies, correcting for imaging-related diagnostic errors, suggests that AF prevalence in type 2 diabetes may be considerably overestimated. Patient flow simulations based on diagnostic accuracy data further indicate that currently recommended screening algorithms may produce high false-positive referral rates with limited sensitivity for detecting true cases—a finding corroborated by recent prospective screening trials. Finally, a review of the available evidence suggests that the clinical, public health and economic benefits of AF screening in type 2 diabetes remain unestablished.

## Diagnosing and staging MASLD in type 2 diabetes

### Approach to diagnosis

Unlike the previous definition of non-alcoholic fatty liver disease (NAFLD), which necessitated exclusion of all other causes of steatosis, MASLD is diagnosed using affirmative criteria (see Text box, ‘Diagnostic criteria for MASLD’) [3]. Diagnosis requires objective evidence of hepatic steatosis in the presence of at least one of five cardiometabolic risk factors, that is, features of the metabolic syndrome [16]. Clinically significant alcohol consumption must be ruled out; this equates to approximately three or more drinks per day for men and two or more drinks per day for women. Individuals exceeding these limits are classified as having either metabolic dysfunction and alcohol-associated liver disease (MetALD) or alcohol-associated liver disease (ALD), depending on the degree of alcohol intake (see Text box). MetALD is an overlap syndrome in which abdominal obesity and excessive alcohol use act as synergistic drivers of disease severity, and it may carry a worse prognosis than MASLD alone [3, 17–20].

**Figure:**
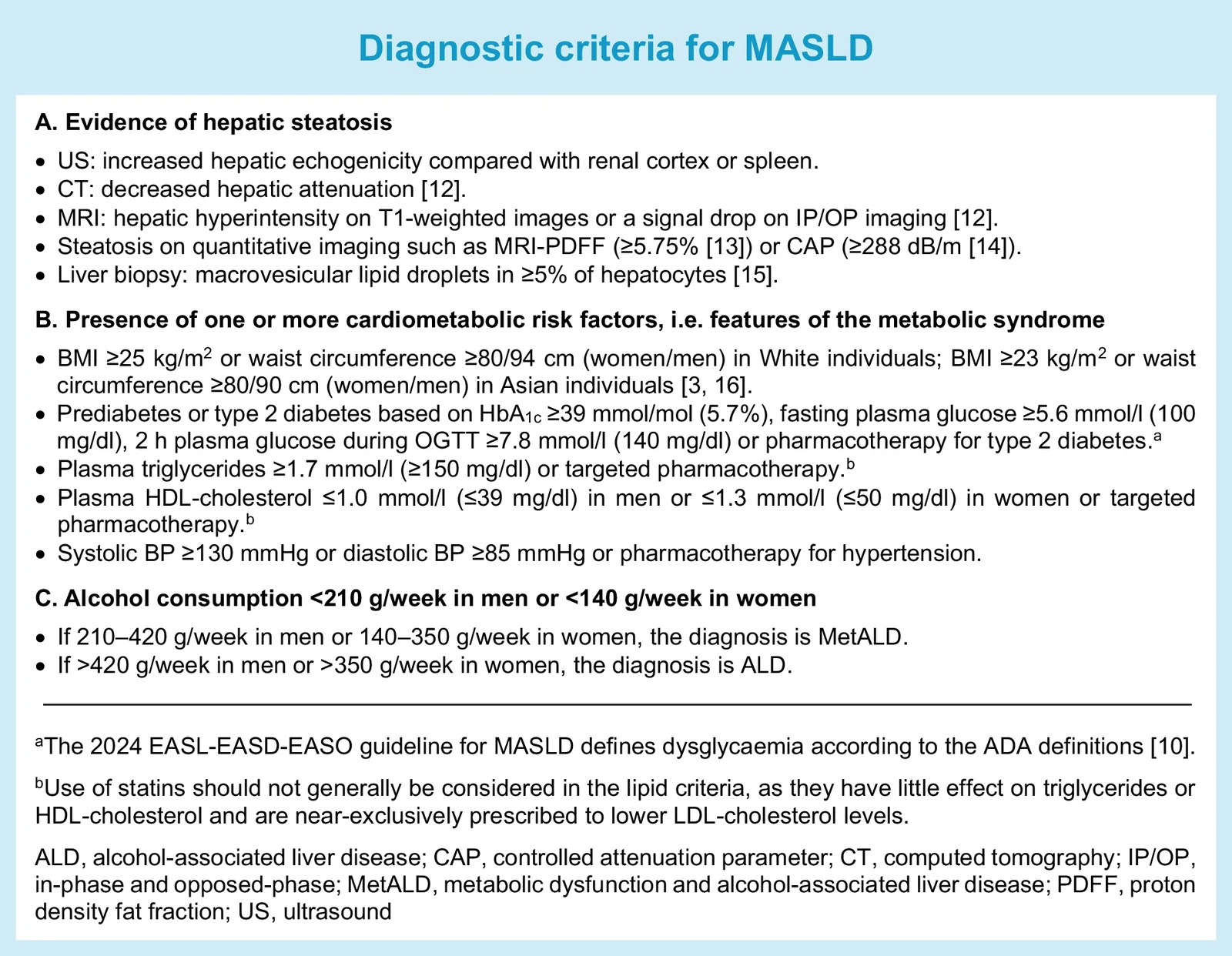

**Fig. 1:**
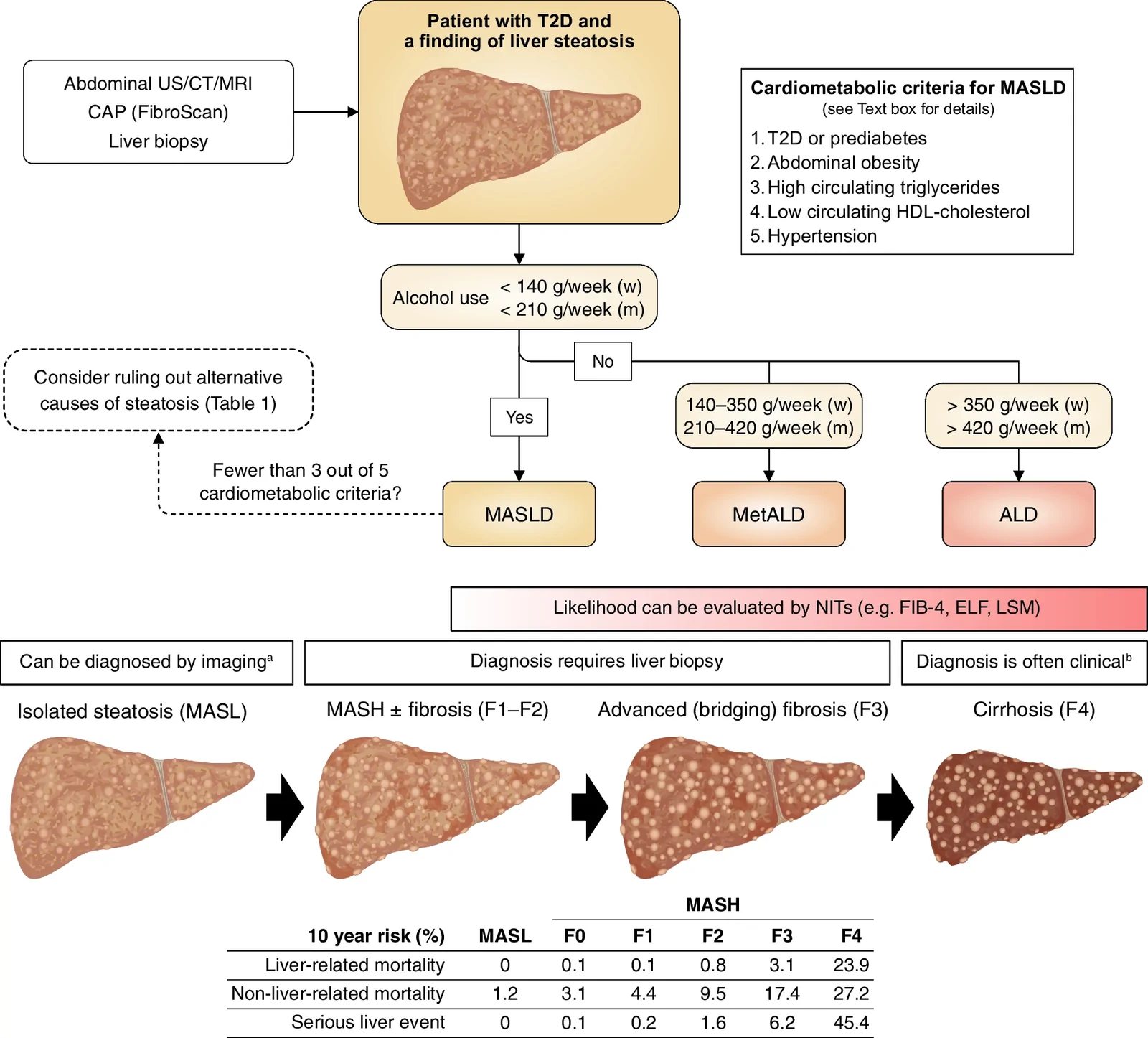
Diagnosing and staging MASLD in individuals with type 2 diabetes. Hepatic steatosis is most often detected incidentally on abdominal imaging. Individuals with type 2 diabetes or prediabetes and evidence of steatosis should first be evaluated for clinically significant alcohol consumption, which may result in a diagnosis of MetALD or ALD depending on the level of intake. Individuals without significant alcohol consumption are classified as having MASLD (see Text box, ‘Diagnostic criteria for MASLD’). The likelihood of MASLD is highest in those meeting criteria for the metabolic syndrome (three or more out of five cardiometabolic risk factors in total, or two or more in addition to type 2 diabetes/prediabetes). In the absence of metabolic syndrome, alternative causes of hepatic steatosis should be considered (Table 1), noting that MASLD may coexist with other specific aetiologies. MASLD spans a spectrum from isolated steatosis (MASL) to MASH with progressive fibrosis and culminating in AF/cirrhosis. Definitive diagnosis of pre-cirrhotic disease requires liver biopsy. However, the likelihood of AF (F3–F4), and to some extent at-risk MASH (MASH + ≥F2), can be assessed using non-invasive tests (NITs). The 10 year risk of liver-related mortality, non-liver-related mortality and serious liver events across the spectrum of MASLD is shown, with values derived for a 60-year-old man using the MASLD Simulator [21]. Serious liver events include liver-related mortality, decompensated cirrhosis and hepatocellular carcinoma. ^a^While steatosis can be diagnosed by imaging across the MASLD spectrum, MASL is a histological diagnosis that precludes steatohepatitis and fibrosis. ^b^The diagnosis of cirrhosis is often based on a constellation of typical clinical, biochemical and radiological findings. CAP, controlled attenuation parameter; CT, computed tomography; ELF, Enhanced Liver Fibrosis test; FIB-4, Fibrosis-4 index; LSM, liver stiffness measurement; m, men; T2D, type 2 diabetes; US, ultrasound; w, women. This figure is available as part of a downloadable slideset

Figure 1 outlines a suggested diagnostic approach to MASLD in people with type 2 diabetes [3, 10]. By definition, all individuals with type 2 diabetes and hepatic steatosis fulfil the MASLD criteria. However, clinicians should remain vigilant for alternative causes of steatosis. There is marked heterogeneity in the pathophysiology of what is termed type 2 diabetes, with many individuals exhibiting predominant insulin deficiency rather than insulin resistance [22]. Because MASLD is specifically associated with insulin-resistant disease endotypes [23, 24], diagnostic consideration should be broadened in those who lack features of the metabolic syndrome (Fig. 1 and Table 1). However, MASLD is highly likely to be the primary underlying aetiology of steatosis if type 2 diabetes is accompanied by at least two other cardiometabolic risk factors [25, 26].

**Table 1:** Differential diagnosis of liver steatosis in individuals with suspected MASLD^a^

Differential diagnosis	Laboratory examinations	Suggestive clinical features
ALD	Screening tests: blood PEth, hair/urine EtG, AST/ALT ratio (>2 is more suggestive of ALD), GGT (disproportionate increase vs ALT and AST in ALD), MCV (macrocytosis in ALD)	History of alcohol use disorder or other substance abuse, history of pancreatitis, signs of malnutrition
Drug-induced liver disease	Laboratory and liver biopsy manifestations are variable and highly dependent on the offending agent. Steatosis may be histologically macrovesicular (as in MASLD) or microvesicular	History of treatment with, for example, methotrexate, glucocorticoids, tamoxifen, oestrogens, amiodarone, 5-fluorouracil, irinotecan, valproate, lomitapide, stavudine or zidovudine
Chronic viral hepatitis (usually HCV genotype 3, rarely HBV)	Screening test: HCV antibodies Confirmatory tests: HCV RNA, HCV genotype	History of i.v. drug use, unprotected sexual intercourse with an infected person, blood transfusions or organ transplants in countries without donor HCV screening
Hypothyroidism	Confirmatory tests: TSH, fT4	Weight gain, fatigue, cold intolerance, bradycardia, hypertension, dyslipidaemia, hyporeflexia, myopathy, constipation, dry and coarse skin, hair loss, infertility
Wilson’s disease	Screening test: serum ceruloplasmin Confirmatory tests: 24 h urinary copper excretion, genetic testing for mutations in *ATP7B*, copper content in liver biopsy	Young age at presentation, neuropsychiatric abnormalities, Kayser–Fleischer rings, Coombs-negative haemolytic anaemia, family history of liver disease and neurological/psychiatric disorders (autosomal recessive inheritance)
Coeliac disease	Screening tests: IgA anti-tissue transglutaminase antibodies (also IgA anti-endomysial and IgA anti-gliadin antibodies) Confirmatory test: duodenal biopsy	Diarrhoea, steatorrhoea, abdominal pain, weight loss, iron deficiency anaemia, deficiency of fat-soluble vitamins, osteoporosis, dermatitis herpetiformis, peripheral neuropathy
Hypobetalipoproteinaemia	Screening tests: plasma triglycerides, cholesterol and ApoB (abnormally low concentrations) Confirmatory test: genetic testing	Steatorrhoea, deficiency of fat-soluble vitamins, acanthocytosis, vision loss, neurological symptoms
Lipodystrophy	Clinical diagnosis with testing dependent on clinical features and suspected aetiology	Generalised or localised lack of s.c. adipose tissue with visceral and skeletal muscle fat accumulation, often severe metabolic syndrome, use of cART (e.g. stavudine or zidovudine) in HIV-associated lipodystrophy

^a^Many other disorders can cause hepatic steatosis under specific circumstances or in isolated cases. This list is not exhaustive but presents the most common or classic causes

ALT, alanine aminotransferase; ApoB, apolipoprotein B; AST, aspartate aminotransferase; cART, combination antiretroviral therapy; EtG, ethyl glucuronide; fT4, free thyroxine; GGT, gamma-glutamyltransferase; HBV, hepatitis B virus; HCV, hepatitis C virus; MCV, mean corpuscular volume; PEth, phosphatidylethanol; TSH, thyroid-stimulating hormone

### Diagnosing steatosis

Owing to the indolent natural history of isolated steatosis and the lack of sufficiently accurate and readily available diagnostic tools, current guidelines do not recommend screening for hepatic steatosis in type 2 diabetes or other populations [10, 11]. Consequently, MASLD is most often identified incidentally on abdominal imaging performed for other clinical indications. Steatosis can be diagnosed based on qualitative radiological features, including increased hepatic echogenicity on ultrasound, decreased attenuation on computed tomography, and signal hyperintensity on MRI [12]. A characteristic imaging feature is focal fatty sparing, where well-defined areas of liver tissue are relatively free of fat accumulation [27].

Because conventional imaging modalities have limited sensitivity for mild steatosis [12, 28], they cannot reliably exclude MASLD, particularly in type 2 diabetes where pre-test probability is high. By contrast, modern MRI scanners now offer the option to quantitatively assess liver fat content via proton density fat fraction (PDFF), a highly sensitive and reproducible method that is considered the gold standard in non-invasive diagnostics [12, 29]. A PDFF of ≥5.75% identifies steatosis with 90% specificity [13]. Controlled attenuation parameter (CAP), an ultrasound-based technique integrated into vibration-controlled transient elastography (VCTE; see ‘Imaging-based NITs’), is an alternative but considerably less accurate method for assessing steatosis quantitatively (CAP ≥288 dB/m is indicative of steatosis) [14, 29, 30].

In liver biopsy, steatosis is defined by the presence of macrovesicular lipid droplets in ≥5% of hepatocytes and is graded as S0 (<5%), S1 (5–33%), S2 (34–66%) or S3 (≥67%) [15]. Of note, these pathologist-reported values are not directly comparable with those obtained by MRI-PDFF; while histological steatosis can in principle reach 100%, PDFF plateaus at approximately 40–50% [13].

### Diagnosing MASH and fibrosis

Although several non-invasive tests (NITs) are available for assessing the likelihood of MASH and especially fibrosis (see ‘NITs for liver fibrosis’), definitive diagnosis requires a liver biopsy (Fig. 1). In MASH, steatosis is accompanied by lobular inflammation and hepatocellular ballooning, all three of which are required for diagnosis [31]. Fibrosis is staged from F0 to F4, with F3 (bridging fibrosis) and F4 (cirrhosis) together referred to as AF [15]. Stage F2 (sinusoidal and portal fibrosis) is often denoted as ‘significant fibrosis’, marking the point at which the risk of progression to cirrhosis, MALOs and mortality begins to increase more markedly (Fig. 1) [32–36].

The term ‘at-risk’ MASH has been adopted to describe MASH with F2/F3 fibrosis, defining the target population for ongoing Phase II–III drug trials in pre-cirrhotic disease [37]. Two compounds, resmetirom and semaglutide, have recently demonstrated positive results in landmark Phase III trials and are now conditionally approved for the treatment of at-risk MASH in both the USA and Europe [7, 8]. Notably, their US Food and Drug Administration (FDA) labels do not require a liver biopsy for diagnosis, facilitating the use of NIT-based approaches to target treatment [38, 39].

### Liver biopsy: its role and limitations

The role of liver biopsy is diminishing, as recent data support the primary use of NITs for risk stratification and guiding treatment decisions [40, 41]. In current practice, biopsy is typically reserved for excluding alternative liver diseases, although it remains the only method for diagnosing MASH and fibrosis definitively (Fig. 1) [10, 11]. Its utility is, however, limited by its high cost, associated discomfort and risk of complications, as well as several important sources of diagnostic error. A percutaneous needle biopsy represents only approximately 1/50,000th of the entire liver volume, introducing significant sampling error and uncertainty in histological diagnosis [42]. There is also marked intra- and inter-rater variability among pathologists in grading key histological features, particularly inflammation and ballooning [43]. These factors greatly undermine the reliability of biopsy not only as a diagnostic tool, but also for monitoring treatment response [44]. Current fibrosis staging is further limited in its ability to resolve clinically meaningful differences in collagen deposition at F3–F4, although recent efforts to increase the granularity of staging may partly address this problem [45]. Finally, the distinction between MASLD, MetALD and ALD rests on clinical criteria and cannot be established on histology alone [46].

## NITs for liver fibrosis

Broadly, NITs can be divided into blood-based and imaging-based NITs. Blood-based NITs include composite risk scores that combine standard laboratory measurements with clinical information (e.g. the Fibrosis-4 index [FIB-4]) or incorporate circulating biomarkers that more directly reflect ongoing fibrogenesis and fibrosis resolution (e.g. the Enhanced Liver Fibrosis [ELF] test). At present, only FIB-4 and ELF have been adopted into routine clinical use [10, 11]. The previously widely used NAFLD Fibrosis Score (NFS) has been found to perform poorly in people with obesity and type 2 diabetes, leading to its elimination from guidelines [47, 48]. Imaging-based NITs include liver stiffness measurement (LSM) using ultrasound-based VCTE or magnetic resonance elastography (MRE), and their availability is often limited to specialist settings. These modalities are more extensively reviewed by Jonuscheit and Schrauwen-Hinderling in this special issue [49].

### Blood-based NITs

#### FIB-4

**Fig. 2:**
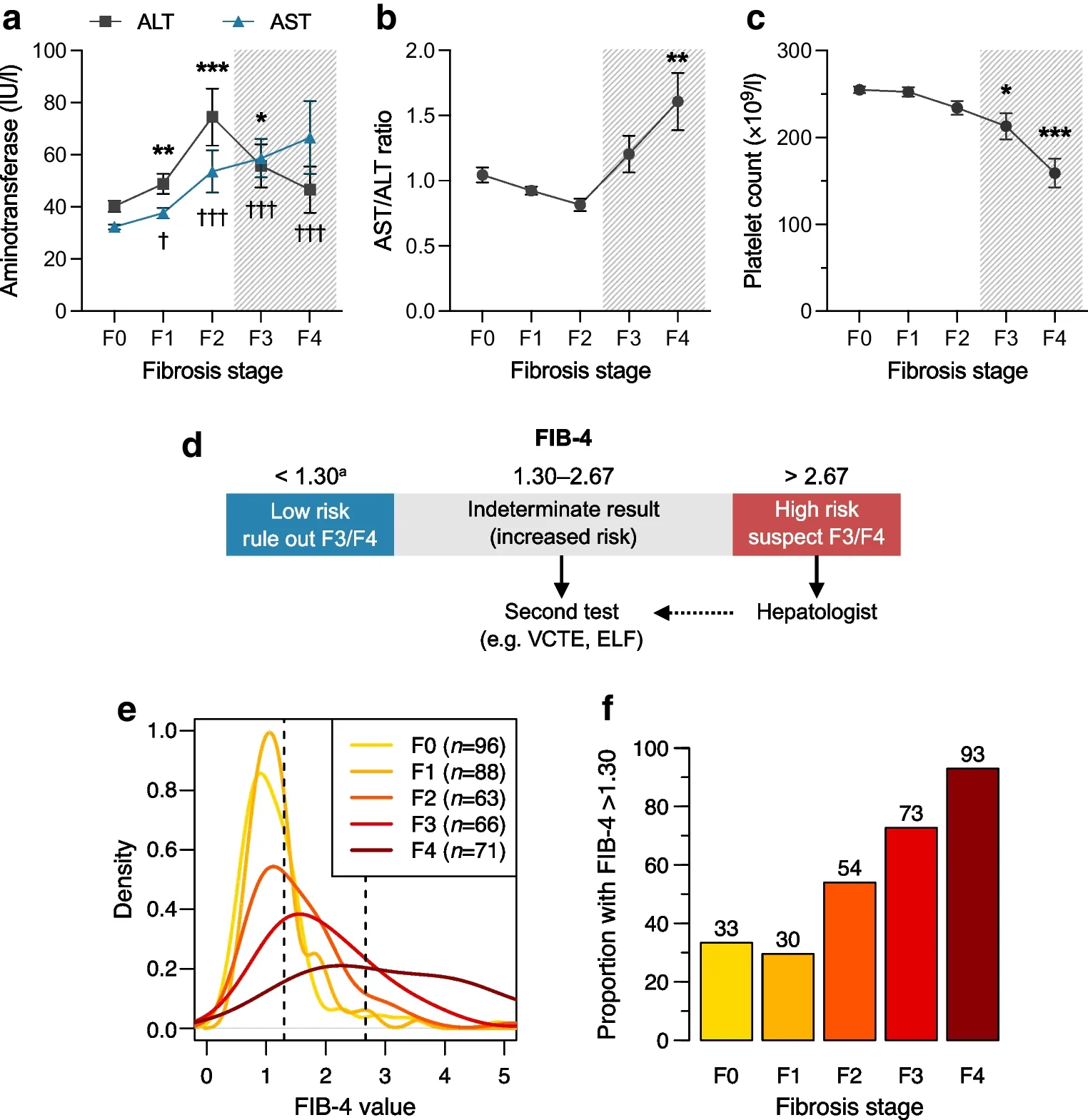
Overview of FIB-4 and its utility in the evaluation of MASLD-related AF. (**a**–**c**) Changes in the FIB-4 components plasma ALT and AST activity (**a**), AST/ALT ratio (**b**) and platelet count (**c**) across histological fibrosis stages in individuals with obesity undergoing liver biopsy (*n*=447; unpublished data from participants in [47]). No participants had decompensated cirrhosis. Shaded areas indicate AF, and data are shown as mean ± SEM. The Kruskal–Wallis test was used with post hoc testing to compare the values of F1–F4 with values of F0. Results were adjusted for multiple comparisons using the two-stage step-up method of Benjamini, Krieger and Yekutieli, as implemented in GraphPad Prism (version 11.0.2 for macOS). Statistical significance is denoted using nominal *p* values as *p≤0.05, **p≤0.01, ***p≤0.001 (for ALT) and ^†^*p*≤0.05, ^†††^*p*≤0.001 (for AST) in (**a**); **p≤0.01 in (**b**); and *p≤0.05, ***p≤0.001 in (**c**). (**d**) Schematic illustration of the use of FIB-4 for estimating the risk of AF. (**e**) Distribution of FIB-4 values within each fibrosis stage in a liver biopsy cohort with type 2 diabetes (*n*=384; unpublished data from participants in [47]). Dashed lines denote the lower (1.30) and upper (2.67) cut-offs of FIB-4. (**f**) Proportion of individuals in each fibrosis stage with FIB-4 values above the lower cut-off (1.30). Individuals with F0–F2 and FIB-4 >1.30 represent false positives when the aim is to identify AF. ^a^A higher rule-out cut-off of 2.0 for FIB-4 has been suggested in those aged ≥65 years [50]. ALT, alanine aminotransferase; AST, aspartate aminotransferase. This figure is available as part of a downloadable slideset

Several commonly measured laboratory parameters show characteristic changes with fibrosis progression. Circulating aminotransferases correlate modestly with liver fat content (*r*=0.3–0.4 [25]) and increase in mild-to-moderate fibrosis (Fig. 2a). With progression to AF, alanine aminotransferase (ALT) activity often declines while aspartate aminotransferase (AST) remains relatively unaffected (Fig. 2a). This divergence leads to a progressive increase in the AST/ALT ratio (Fig. 2b), even though absolute aminotransferase levels may eventually normalise [51]. A decreased platelet count is another hallmark of AF, with thrombocytopenia in cirrhosis arising through multiple mechanisms (Fig. 2c) [52].

FIB-4 is a fibrosis risk score calculated from age, ALT, AST and platelet count, which are parameters often routinely measured at type 2 diabetes follow-up (Table 2). It is the most widely validated NIT in MASLD and, among simple risk scores, performs most consistently across diverse populations and clinical settings [4, 10, 11, 57]. Unlike many other NITs, its performance is not adversely affected by obesity [47]. The simplicity of FIB-4 enables automated calculation within electronic medical records, which may support broader use in routine care [58]. In addition to identifying AF, FIB-4 has also been shown to predict fibrosis progression and the risk of liver-related adverse outcomes, with a similar performance to that of liver biopsy [59–61]. These attributes have led to its adoption as the preferred first-line NIT [4, 10, 11].

**Table 2:** Diagnostic performance of blood-based and imaging-based NITs for the assessment of AF

Test	Formula or method	AUROC for AF	Cut-off for AF^a^	Sensitivity (95% CI)	Specificity (95% CI)	Population (T2D/mixed)	Reference
FIB-4	$\frac{\mathrm{Age}(\mathrm{years})\times\mathrm{AST}(\mathrm{IU}/\mathrm l)}{\mathrm{Platelets}(\mathrm E9/\mathrm l)\times\sqrt{\mathrm{ALT}(\mathrm{IU}/\mathrm l)}}$	0.75	1.30	73 (69, 76)	62 (59, 65)	T2D	[53]
			2.67	23 (20, 26)	93 (92, 95)		
ELF	Proprietary blood test analysing HA, PIIINP and TIMP-1	0.83	7.7	93 (82, 98)	34 (13, 65)	Mixed	[54]
			9.8	65 (49, 77)	86 (77, 92)		
			11.3	36 (15, 63)	96 (90, 99)		
LSM-VCTE	Ultrasound-based method; proprietary hardware and software	0.82	8.0 kPa	88 (86, 90)	56 (53, 60)	T2D	[53]
			9.7 kPa	77 (74, 80)	71 (68, 74)		
			12.1 kPa	55 (52, 57)	90 (89, 91)	Mixed	[55]
LSM-MRE	MRI-based method; proprietary hardware and software	0.92	3.53 kPa^b^	87 (80, 92)	88 (81, 94)	Mixed	[56]

All of the diagnostic accuracy studies cited here used liver biopsy as the reference standard

^a^For a given cut-off, values equal to or greater than the cut-off are considered positive, and values less than the cut-off are considered negative

^b^The 3.53 kPa cut-off for LSM-MRE was optimised in the latest individual participant data meta-analysis by Liang et al [56]. A cut-off of 3.62 kPa has been widely used to identify AF, and it had a sensitivity of 78% (95% CI 66, 88) and specificity of 88% (95% CI 82, 92) in the same study

ALT, alanine aminotransferase; AST, aspartate aminotransferase; AUROC, area under the receiver operating characteristic curve; HA, hyaluronic acid; PIIINP, procollagen III amino-terminal peptide; TIMP-1, tissue inhibitor of matrix metalloproteinase 1; T2D, type 2 diabetes

As with most NITs, two thresholds are applied for FIB-4 when using it in clinical decision making (Fig. 2d). The upper threshold of 2.67 has a specificity of 93% (95% CI 92, 95) and identifies those who require further evaluation, but its sensitivity is low and up to 80% of individuals with AF fall below this level. The lower threshold of 1.30 has been adopted for ruling out AF and has a sensitivity of 73% (95% CI 69, 76) (Fig. 2d, Table 2). However, a quarter of AF cases have FIB-4 <1.30 and will therefore be misclassified as low risk. Another major challenge is that at least 30–40% of tested individuals fall into the indeterminate range (1.30–2.67) and therefore require a second, more accurate NIT (Fig. 2d). These limitations reflect the modest diagnostic ability of FIB-4, illustrated by the substantial overlap in its values across fibrosis stages (Fig. 2e). Furthermore, one-third of individuals with F0–F1 and half of those with F2 may have FIB-4 >1.30, representing false positives from the standpoint of AF identification (Fig. 2f).

FIB-4 may be falsely elevated in the presence of thrombocytopenia from non-hepatic causes or pseudothrombocytopenia due to in vitro platelet aggregation. It also tends to underestimate fibrosis risk in younger people while overestimating it in older individuals [50]. To maintain specificity in those aged ≥65 years, a higher rule-out threshold of 2.0 is recommended, although it has not been widely validated [50]. Conversely, the performance of FIB-4 in individuals aged <35 years is difficult to assess, as AF is uncommon in this age group and therefore poorly represented in liver biopsy cohorts [50]. Finally, evidence is conflicting as to whether the diagnostic performance of FIB-4 is diminished in type 2 diabetes [53, 62–65]. Some investigators have proposed that more specialised blood-based or composite NITs, such as FibroMeter or Agile-3+, should be prioritised as first-line tools in this population [62, 63].

#### ELF

In contrast to FIB-4, the ELF test is a patented commercially available NIT (Siemens Healthineers, Forchheim, Germany) that is based on serum concentrations of three extracellular matrix components (Table 2). It has been extensively validated across diverse populations, including people with obesity and type 2 diabetes [4, 10, 11, 57, 66, 67]. Similar to FIB-4, ELF predicts future liver-related outcomes [68]. ELF <7.7 rules out AF with a sensitivity of 93% (95% CI 82, 98), whereas ELF ≥9.8 rules in AF with a specificity of 86% (95% CI 77, 92) (Table 2). An even higher threshold of ≥11.3 is predictive of cirrhosis and is associated with a high 5 year risk of liver-related clinical events [69].

While FIB-4 is derived from routine laboratory measurements, which are often available at no additional cost, ELF is substantially more expensive, making it unsuitable as a first-line test. However, its cost is comparable to that of VCTE and, although few head-to-head comparisons exist, ELF may have a similar or slightly inferior diagnostic performance. The Liver Investigation: Testing Marker Utility in Steatohepatitis (LITMUS) study reported areas under the receiver operating characteristic curve (AUROC) for detecting AF of 0.80 (95% CI 0.76, 0.83) for ELF and 0.77 (95% CI 0.74, 0.81) for FIB-4 (*n*=919) [65]. In a partially overlapping subsample (*n*=632), LSM-VCTE achieved an AUROC of 0.83 (95% CI 0.80, 0.86). Most guidelines currently recommend ELF as an alternative second-line test to VCTE [10, 11]. As a standardised blood-based NIT, ELF is operator independent and not subject to measurement failure, which can occur with VCTE, especially in individuals with obesity [70]. ELF may also be helpful in decision making when other NITs yield discordant or indeterminate results [57]. Importantly, its performance appears relatively unaffected by age, making it a potentially suitable option in individuals aged <35 or ≥65 years [67, 71].

#### Other blood-based NITs

Although many simple scores have been developed for assessing AF risk, none have shown significant advantages over FIB-4 in large validation studies [65]. Efforts to generate improved algorithms from commonly available markers are ongoing, for instance by leveraging machine learning [72], although they require further validation. To meaningfully improve on FIB-4 will likely require specialised NITs that incorporate circulating biomarkers of fibrogenesis, similar to ELF. The LITMUS study found that, of 17 NITs evaluated, only the ADAPT score (based on PRO-C3, a marker of type III collagen formation) and SomaSignal (a proteomics-based score) surpassed the predefined acceptable AUROC of 0.80 for AF [65]. Other specialised NITs not included in that study, such as the recently developed FIB-11 (which combines nine common markers with hyaluronate and alpha-2-macroglobulin), show promise for increasing accuracy and markedly reducing the indeterminate zone between rule-in and rule-out thresholds [72].

The importance of at-risk MASH as an indication for newly approved pharmaceuticals has prompted development of NITs to better identify it, as FIB-4 and other AF tests perform poorly for this purpose [65, 73]. The Fibrotic NASH Index (FNI) is a simple score based on AST, HDL-cholesterol and HbA_1c_ that outperforms FIB-4 for identifying at-risk MASH, including in individuals with type 2 diabetes [74–76]. Specialised NITs such as MACK-3 [77] (combining glucose, insulin, AST and CK-18) and NIS4 [78] (combining HbA_1c_ with four specialised biomarkers) may achieve even greater accuracy, and the latter has been validated in the Non-Invasive Biomarkers for Metabolic Liver Disease (NIMBLE) study for predicting at-risk MASH, with a high AUROC of 0.81 (95% CI 0.78, 0.84) [73].

### Imaging-based NITs

#### LSM by VCTE and MRE

LSM is the most direct method of assessing fibrosis non-invasively, as it reflects the physical changes that occur in liver during fibrosis progression. The most widely adopted and validated NIT for LSM is ultrasound-based VCTE (FibroScan, Echosens, Paris, France). It is a portable point-of-care device that has undergone extensive histological validation and is optimised for reproducible measurements by trained operators [57]. An LSM-VCTE <8.0 kPa rules out AF with a sensitivity of 88% (95% CI 86, 90), whereas an LSM-VCTE ≥12.1 kPa rules in AF with a specificity of 90% (95% CI 89, 91) (Table 2). The 9.7 kPa threshold provides a more balanced sensitivity (77% [95% CI 74, 80]) and specificity (71% [95% CI 68, 74]). Two probes are available (M and XL), with the XL probe required in individuals with increased skin-to-capsule distance, as commonly seen in obesity. Similar diagnostic thresholds apply to both probes [79]. LSM-VCTE and its composite scores (see ‘Composite blood- and imaging-based NITs’) have good prognostic utility in MASLD, equalling and potentially even exceeding that of liver biopsy [60, 61, 80–83].

Measurement failure is a key limitation of VCTE, occurring in ≥20% of examinations with the M probe in populations with a high obesity prevalence, but decreasing to <10% when use of either probe is possible [84]. Despite built-in standardisation, VCTE remains operator dependent, and inexperienced users with less than 100–500 measurements have lower success rates and produce less accurate measurements [85, 86]. False positives may also occur with food intake within 2–3 h of examination, excessive alcohol intake, acute hepatitis, congestive heart failure and extrahepatic cholestasis [57].

MRE represents the current state of the art for LSM, using conventional MRI to quantify the propagation of externally applied shear waves through liver tissue [87]. Despite full commercialisation, its use remains largely confined to university hospitals and research settings. A recent meta-analysis showed that an LSM-MRE ≥3.53 kPa identifies AF with an AUROC of 0.92 (95% CI 0.90, 0.94), resulting in a high sensitivity (87% [95% CI 80, 92]) and specificity (88% [95% CI 81, 94]) (Table 2) [56].

### Composite blood- and imaging-based NITs

Several composite NITs incorporate LSM alongside blood-based markers with the aim of further improving diagnostic accuracy. The Agile-3+ and Agile-4 scores combine LSM-VCTE with AST, ALT, platelet count, sex and type 2 diabetes status to identify AF and cirrhosis, respectively, outperforming FIB-4 (although not necessarily LSM alone) in individuals with type 2 diabetes [63, 88]. For prognostication, however, they outperform FIB-4 and other simple NITs, LSM alone, the FAST score and even histological fibrosis staging [80, 83]. The FAST and MAST scores pair LSM-VCTE or LSM-MRE, respectively, with CAP and AST (FAST) or MRI-PDFF and AST (MAST) to identify at-risk MASH, outperforming LSM alone as well as simple blood-based NITs such as FIB-4 and FNI [74, 89].

## Guideline recommendations for fibrosis screening in people with type 2 diabetes

### Rationale for screening

**Fig. 3:**
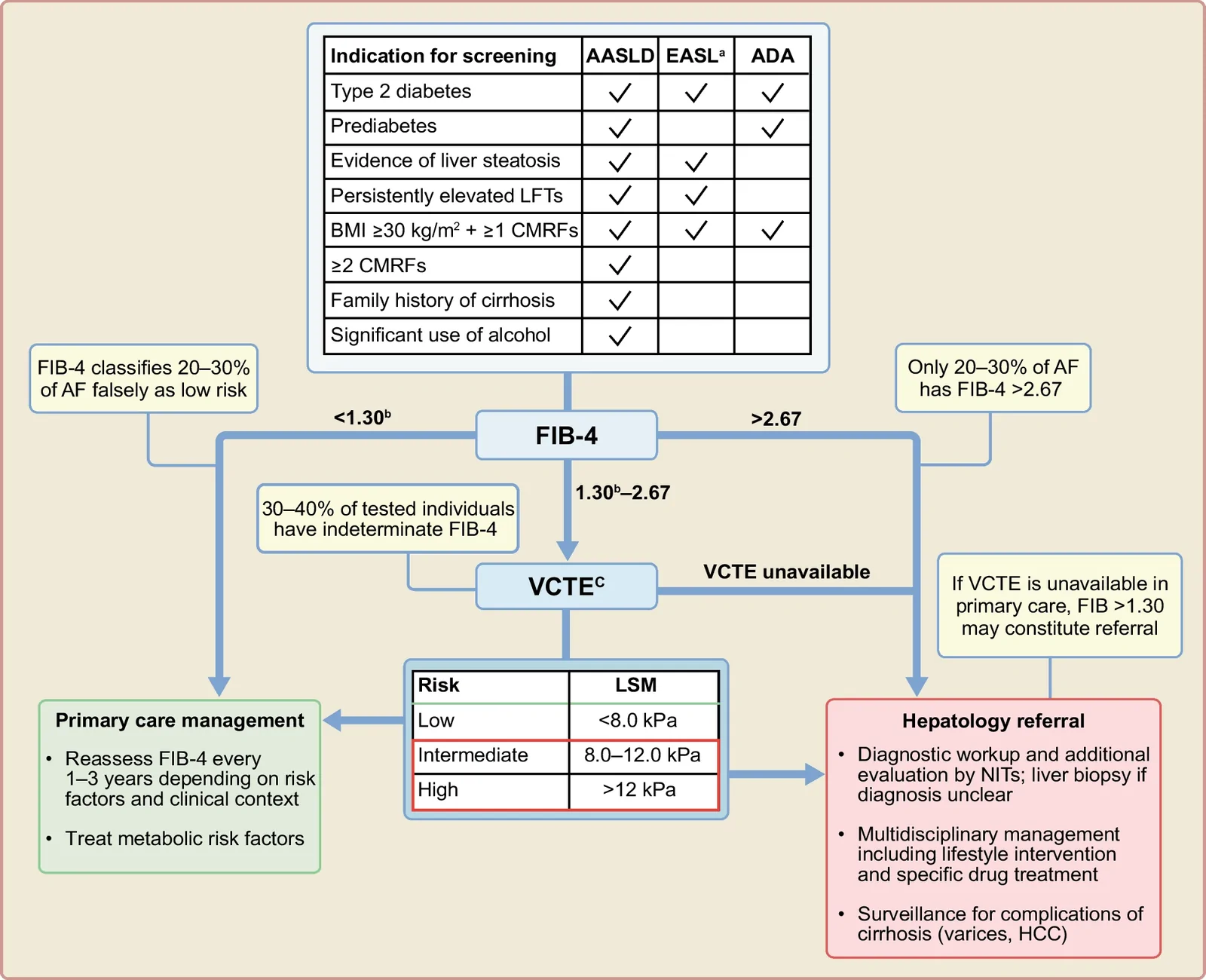
Algorithm for screening for MASLD-related AF in primary care and diabetology clinics. Indications for fibrosis screening vary across guidelines, but all major societies recommend screening for AF in individuals with type 2 diabetes, irrespective of a prior MASLD diagnosis. The AASLD and ADA also extend screening to individuals with prediabetes. ^a^Joint EASL-EASD-EASO guideline. ^b^A FIB-4 rule-out threshold of 2.0 has been proposed for individuals aged ≥65 years. ^c^If VCTE is unavailable, an ELF ≥9.8 may be used instead to guide referral, although this threshold may not match the diagnostic performance of LSM-VCTE ≥8.0 kPa. CMRF, cardiometabolic risk factor; HCC, hepatocellular carcinoma; LFT, liver function test. This figure is available as part of a downloadable slideset

A major shift in MASLD clinical practice guidelines occurred in 2023, beginning with updated recommendations from the AASLD [11] and soon followed by similar guidance from the ADA [4, 90] and jointly from the EASL, EASD, and EASO [10]. A key departure from previous guidance was the endorsement of systematic screening for MASLD-related AF in selected populations deemed to be at high risk. Although target groups differ across guidelines, people with type 2 diabetes are consistently identified as a priority population for fibrosis screening (Fig. 3). Notably, the 2018 AASLD guidelines explicitly advised against such screening, citing insufficient data regarding biomarker performance, therapeutic options, clinical outcome benefit and cost-effectiveness [91].

According to these societies, the rationale for screening is supported by accumulating evidence linking type 2 diabetes to a high burden and accelerated progression of liver disease [11]. Compared with individuals without diabetes, in individuals with type 2 diabetes hepatic steatosis is two- to threefold more prevalent [2], fibrosis progression may be ~40% faster [92] and the 10 year risk of MALOs is twofold greater in relative terms [93]. In addition, type 2 diabetes is associated with a severalfold increased risk of hepatocellular carcinoma (HCC), independent of other metabolic risk factors [94]. A central driver of the advocacy for screening has been the emergence of studies suggesting an exceedingly high AF prevalence in type 2 diabetes of up to 20–40% [10, 11, 90]. However, as discussed in the following section, these figures may be markedly overestimated.

### Recommended algorithm for identifying MASLD-related AF

**Fig. 4:**
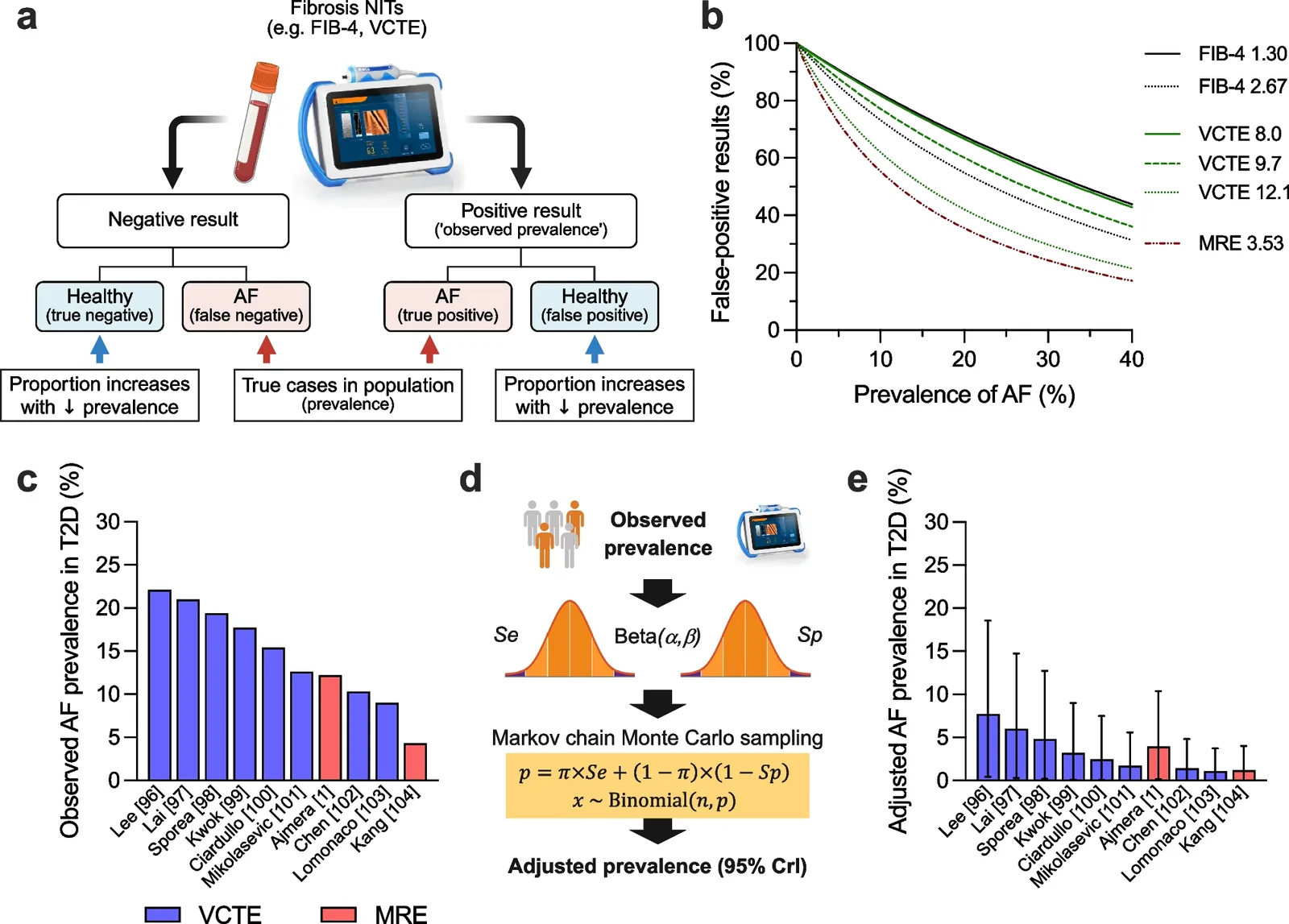
Influence of AF prevalence on the false discovery rate of NITs and implications for LSM-based prevalence estimates. (**a**) Positive/negative NIT results always represent a mixture of true and false positives/negatives. When AF is rare in the tested population, a large proportion of positive results will be false positives, affecting both clinical interpretation and attempts to estimate AF prevalence from NIT positivity. (**b**) Effect of AF prevalence on the proportion of positive test results that are false positives (i.e. false discovery rate [FDR]) for FIB-4, LSM-VCTE and LSM-MRE at commonly used cut-offs (Table 2), calculated as $\mathrm{F}\mathrm{D}\mathrm{R}=\frac{(1-Sp)\times (1-\pi )}{\left(1-Sp\right)\times \left(1-\pi \right)+Se\times \pi }$, where Se and Sp are the sensitivity and specificity of the NIT, respectively, and π is the prevalence of AF. (**c**) Observed AF prevalence defined by LSM-VCTE (cut-offs 9.6–9.7 kPa) or MRE (cut-offs 3.60–3.63 kPa) in studies of type 2 diabetes populations (data from Table 1 in [95]) [1, 96–104]. (**d**) Illustration of a simple Bayesian modelling framework that can be used to adjust observed prevalences (p) to account for the error associated with the use of NITs (as described in [105]). Prior probability distributions for the Se and Sp of each NIT were specified as beta distributions, parameterised to reflect the author’s evaluation of the likely range of plausible values. Markov chain Monte Carlo sampling was then performed, relating the number of positive test results (x) among n tested individuals to p through a binomial likelihood, to derive the posterior distribution of the adjusted (‘true’) prevalence (π) and its 95% credible interval (CrI). Further details are provided in ESM Methods. (**e**) Adjusted AF prevalence estimates and 95% CrIs, obtained by applying the Bayesian framework in (**d**) to the observed prevalences in (**c**). The adjusted values depend on the assumed prior distributions for NIT Se/Sp and should therefore be interpreted as illustrative of the potential magnitude of error in NIT-based prevalence studies rather than as definitive estimates of true AF prevalence. This figure is available as part of a downloadable slideset

Figure 3 illustrates the screening algorithm for MASLD-related AF that is broadly shared across recent guidelines. Primary risk assessment with FIB-4 is recommended for all people with type 2 diabetes. A FIB-4 <1.30 indicates a low likelihood of AF, and these individuals can be managed in primary care with repeat testing every 1–3 years. If FIB-4 is ≥1.30, a more accurate second-line NIT should be performed, preferably VCTE, and those with LSM ≥8.0 kPa should be referred to a hepatologist. A FIB-4 >2.67 may prompt direct referral, although VCTE should still be performed given the limited positive predictive value of this threshold in low-prevalence populations (Fig. 4b). The EASL-EASD-EASO guideline uniquely offers an alternative pathway for individuals with FIB-4 1.30–2.67: depending on medical history, clinical context and local resources, intensified management of comorbidities may be undertaken in lieu of immediate VCTE, with FIB-4 reassessed within 1 year and VCTE performed if FIB-4 remains ≥1.30. It is important to note that, when VCTE is not available outside tertiary care, FIB-4 ≥1.30 effectively constitutes hepatology referral. Some guidelines recommend ELF as an alternative second-line NIT [10, 11]. However, none of the currently validated ELF cut-offs directly corresponds to an LSM of 8.0 kPa by VCTE, and these thresholds are thus likely to evolve (Table 2) [4]. Values in the intermediate range (7.7–9.7) largely fall within the reference interval for healthy individuals and do not confer an increased risk of liver-related events [106]. At present, ELF ≥9.8 is the most practical threshold for referral [4], but clinicians should recognise its potentially limited sensitivity (~65%).

## Pre-test probability: prevalence of AF in type 2 diabetes

### Influence of prevalence on NITs

When evaluating the performance of a NIT in a clinical context—or indeed a screening pathway for AF—an accurate estimate of disease prevalence in the target population is essential [107]. Pre-test probability directly determines the predictive values of an NIT by influencing the proportion of false-positive and false-negative results (Fig. 4a). When prevalence is low, even tests with modest sensitivity can effectively rule out disease, but positive results are more likely to be false positives. Conversely, as prevalence increases, a greater proportion of positive results will be true positives. Figure 4b illustrates how AF prevalence affects the expected proportion of false-positive results for different thresholds of FIB-4 and LSM by VCTE or MRE. This relationship is particularly relevant for screening, where first-line tests are typically simple and widely available but have limited diagnostic accuracy (i.e. FIB-4). If prevalence is lower than anticipated, large numbers of false positives may be referred for costly follow-up investigations and tertiary care.

### Unsuitability of liver biopsy for estimating AF prevalence

Establishing true AF prevalence in type 2 diabetes is complicated by the fact that fibrosis stage can be definitively established only by liver biopsy, which itself is an imperfect reference standard (see ‘Liver biopsy: its role and limitations’). Because a biopsy cannot be ethically performed without sufficient evidence of underlying liver disease, studies with histological data will inherently overestimate AF prevalence. To this end, a meta-analysis of ten studies including 740 participants (range 16–151) with type 2 diabetes undergoing liver biopsy found a pooled AF prevalence of 15% (95% CI 7, 31) [2]. Recently, Castera et al reported a prospective study of 330 participants with type 2 diabetes who underwent liver biopsy, out of 713 individuals referred from diabetology clinics to hepatology for suspected MASLD [108]. In this biopsy cohort, MASH was diagnosed in 58% of participants and AF in 38%. However, the cohort was selected from individuals with ultrasound evidence of hepatic steatosis and persistently elevated ALT. The patients also had advanced type 2 diabetes, reflected by a median disease duration of 9 years (IQR 5–15), microvascular complications in 60%, macrovascular complications in 20% and insulin use in 43% [108]. It is also unknown how many patients diabetologists screened before the 713 were selected for referral. Thus, while the study commendably highlights a high risk of AF in a particular subgroup of individuals with advanced type 2 diabetes, selection bias limits its generalisability to the broader type 2 diabetes population.

### Imaging tests overestimate AF prevalence

To circumvent the bias associated with liver biopsy, a series of studies have attempted to estimate AF prevalence in type 2 diabetes using LSM (see Table 1 in [95]) [1, 96–104]. These studies defined AF by LSM values exceeding prespecified thresholds, yielding prevalence estimates of 4–22% (Fig. 4c) [95]. At face value, these data appear to support the notion of a high AF burden in type 2 diabetes. However, disease prevalence cannot be inferred directly from positive NIT results [109]. This is because positive results always reflect a mixture of true and false positives and, as underlying prevalence decreases, the proportion of false positives increases (Fig. 4a, b) [107], leading to substantially inflated prevalence estimates.

A defined mathematical relationship links test sensitivity (Se) and specificity (Sp), the observed proportion of positive results (p) and the true disease prevalence (π) [109]. When the first three are known, prevalence can in principle be calculated from a simple equation ($\pi =\frac{p+Sp-1}{Se+Sp-1}$). For example, Ajmera et al reported an AF prevalence of 12.2% in 418 adults with type 2 diabetes assessed using state-of-the-art MRE (LSM ≥3.63 kPa) [1]. Assuming sensitivity and specificity of 90% for MRE, values that are higher than those reported in individual participant data meta-analyses [56, 110, 111], the true prevalence in this cohort would be only 2.8%. A limitation of this approach is that the sensitivity and specificity of any given test can vary significantly across studies due to the spectrum effect [112]. A more robust solution is probabilistic Bayesian modelling, in which NIT sensitivity and specificity are assigned prior probability distributions based on the best available evidence from diagnostic accuracy studies (Fig. 4d; see electronic supplementary material [ESM] Methods for further details) [105]. Applying this method to LSM-based AF prevalence estimates yields adjusted prevalences of between 1% and 8% in individuals with type 2 diabetes (Fig. 4e).

These considerations suggest that AF prevalence in type 2 diabetes may be substantially overestimated, which has major implications for the expected performance and justification of fibrosis screening in this population. They also underscore the importance of accounting for diagnostic error when interpreting NIT-based prevalence studies.

## Predicted and real-world outcomes of fibrosis screening in type 2 diabetes

### Predicted outcomes of AF screening

**Fig. 5:**
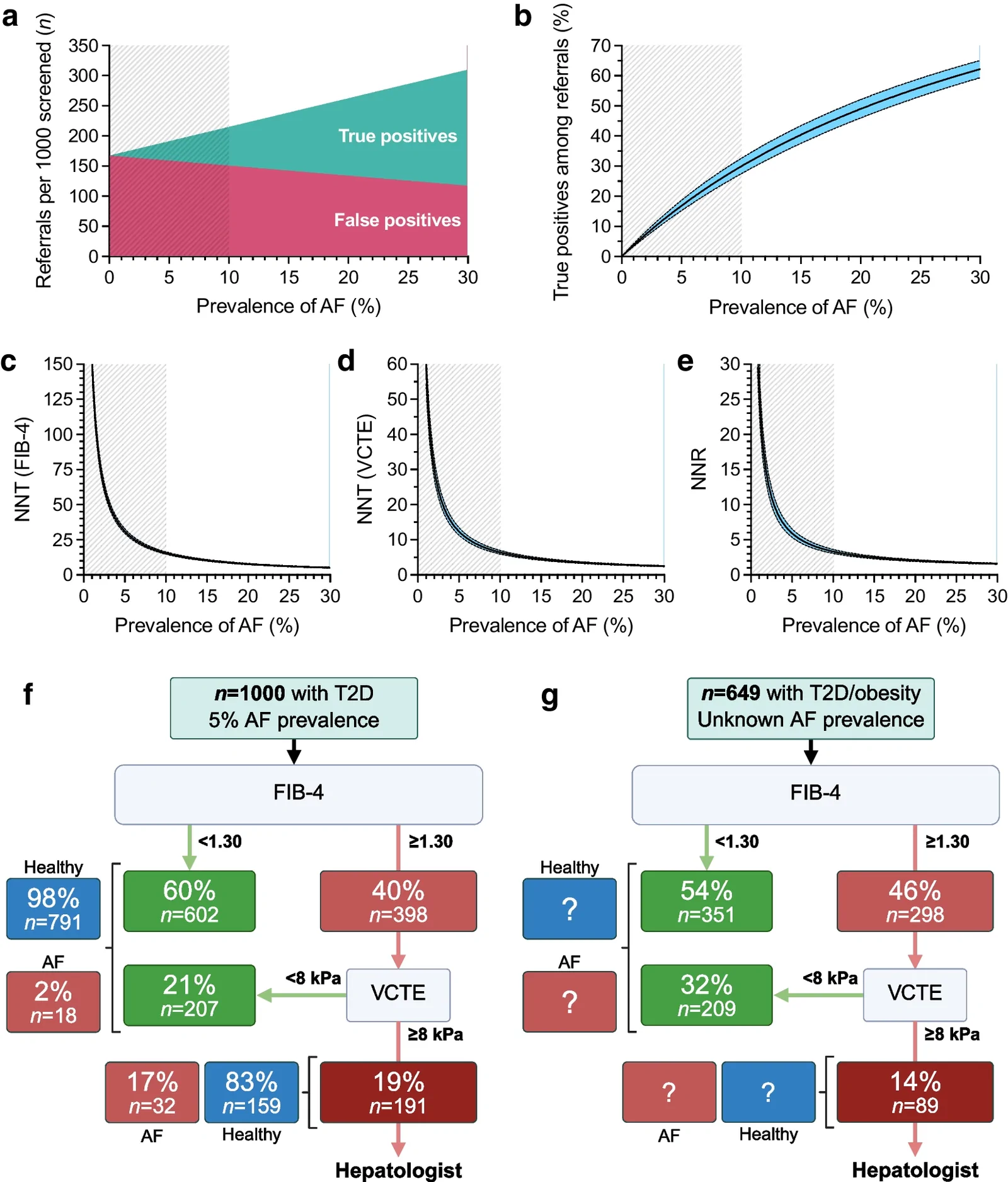
Predicted and real-world outcomes of screening for AF in type 2 diabetes according to current guidelines. (**a**–**e**) Predicted performance metrics for a two-step AF screening pathway starting with FIB-4 and followed by VCTE for all those with FIB-4 ≥1.30 (as shown in Fig. 3). The calculations are based on sensitivities/specificities for FIB-4 1.30 and VCTE 8.0 kPa cut-offs as outlined in Table 2. The following formulas were used [113]: ${Se}_{Tot}={Se}_{FIB4}\times {Se}_{VCTE}$; ${Sp}_{Tot}={1-(1-Sp}_{FIB4})\times (1-{Sp}_{VCTE})$; $TPrate=\pi \times {Se}_{Tot}$; $FPrate=\left(1-\pi \right)\times \left(1-{Sp}_{Tot}\right)$; $VCTErate=\pi \times {Se}_{FIB4}+\left(1-\pi \right)\times \left(1-{Sp}_{FIB4}\right)$; REFrate=TPrate+FPrate; ${PPV}_{Tot}=\frac{TPrate}{REFrate}$; ${NNT}_{FIB4}=\frac{1}{TPrate}$; ${NNT}_{VCTE}=\frac{VCTErate}{TPrate}$; $NNR=\frac{1}{{PPV}_{Tot}}$; $True\,positives / 1000=1000\times TPrate$; $False \,positives / 1000=1000\times FPrate$, where ${Se}_{Tot}$/${Sp}_{Tot}$ is the overall sensitivity/specificity of the screening pathway assuming conditional independence of FIB-4 and VCTE; ${Se}_{FIB4}$/${Sp}_{FIB4}$ and ${Se}_{VCTE}$/${Sp}_{VCTE}$ are the sensitivities/specificities of FIB-4 and VCTE; TPrate is the proportion of a screened population with prevalence π that will be true positives; FPrate is the proportion that will be false positives; VCTErate is the proportion that will require VCTE; REFrate is the proportion that will require hepatology referral; ${PPV}_{Tot}$ is the positive predictive value of the screening pathway (i.e. proportion of true positives among referrals); ${NNT}_{FIB4}$ (**c**) and ${NNT}_{VCTE}$ (**d**) are the numbers needed to test with FIB-4 and VCTE to refer one true case of AF to hepatology; and NNR (**e**) is the number of referrals required to refer one true case of AF to hepatology. It is assumed that VCTE is performed at the primary care level before hepatology referral. To the extent that FIB-4 and LSM-VCTE are positively correlated, these calculations may underestimate ${Se}_{Tot}$ while overestimating ${Sp}_{Tot}$ [114]. For panels (**b**–**e**), 95% CIs were derived by Monte Carlo simulation with 10,000 draws, with sensitivities and specificities of FIB-4 and VCTE sampled independently from beta distributions fitted to their reported point estimates and 95% CIs (Table 2). The shaded areas indicate an AF prevalence level (0–10%) at which screening pathway performance deteriorates markedly. (**f**) Simulated patient flow for a hypothetical cohort of 1000 patients with type 2 diabetes with 5% assumed AF prevalence. Those labelled as healthy do not have AF, but some may have F1/F2 fibrosis. (**g**) Real-world data from a prospective screening study by Caussy et al [115]. True disease status is unknown for participants in this study because liver biopsy was performed only in a small subsample. This figure is available as part of a downloadable slideset

A substantial body of evidence describes the performance of fibrosis NITs in MASLD and type 2 diabetes, which allows estimation of the likely outcomes of AF screening algorithms. Figure 5a–e shows simulations of key performance parameters for the recommended screening pathway (Fig. 3) across AF prevalence values from 0% to 30%. Within this prevalence range, 170–310 of 1000 screened individuals are expected to require hepatology referral (Fig. 5a). The proportion of false positives among those referred depends strongly on the underlying prevalence of AF, exceeding 50% when the prevalence is below 20% (Fig. 5a, b). For both FIB-4 and VCTE, the number needed to test (NNT) to identify a single case of AF for referral rises sharply as prevalence falls into the sub-10% range, accompanied by a marked increase in the number needed to refer (NNR) (Fig. 5c–e). These metrics underscore the importance of having an accurate estimate of true AF prevalence when assessing the feasibility of screening in a given target population.

Figure 5f illustrates the simulated patient flow when screening 1000 individuals with type 2 diabetes for AF, assuming a prevalence of 5%. Of these individuals, 398 require VCTE and 191 are ultimately referred to hepatology. Among those referred, 159 (83%) are false positives and only 32 (17%) truly have AF, yielding an NNR of 6. In practical terms, five healthy individuals are referred for every true case of AF, requiring further evaluation with additional NITs or liver biopsy. These estimates assume that VCTE is available in primary care. If not, all 398 individuals with FIB-4 ≥1.30 would require a hepatology referral, increasing the NNR to 13 (i.e. the NNT for VCTE at 5% prevalence; Fig. 5d). Notably, of the 50 individuals with AF, 18 (36%) would be misclassified as low risk within this pathway.

### Real-world evidence from prospective screening studies

Interim results from a prospective multicentre study by Caussy et al provide real-world support for these projections [115]. Individuals with type 2 diabetes and/or obesity were screened in university hospital diabetology and nutrition clinics according to current guidelines, with the resulting participant flow shown in Fig. 5g. Among 649 participants, 298 (46%) required VCTE and 89 (14%) a hepatology referral. Because liver biopsy was performed only when clinically indicated, the true disease status of those referred or classified as low risk cannot be determined. Of the 52 participants who underwent biopsy, 24 (46%) had AF, corresponding to 3.6% of the overall cohort. An additional 5.6% had LSM values suggestive of AF by MRE (>3.6 kPa) or VCTE (≥12 kPa). However, depending on the underlying prevalence of AF, some of these cases could be false positives (Fig. 4b). Similar VCTE and hepatology referral rates have been reported in large retrospective studies evaluating AF screening algorithms in individuals with type 2 diabetes [116–118].

In another screening study from Sweden, Lindfors et al offered VCTE to individuals with type 2 diabetes attending routine retinopathy surveillance, referring those with an LSM ≥8.0 kPa to hepatology [119]. Of 977 participants with reliable measurements, 154 (16%) had an LSM ≥8.0 kPa and 49 (5%) had an LSM >12.0 kPa. However, at repeat VCTE after a median of 39 days, 56 of the 124 re-tested participants (45%) had an LSM <8.0 kPa, suggesting a high false-positive referral rate. LSM was systematically higher when measured by trained nurses at screening than by hepatologists at follow-up, which the authors attributed to factors such as operator inexperience, potential behavioural changes (e.g. alcohol abstinence following an abnormal result) and better adherence to fasting instructions at the second visit [119].

The LiverScreen consortium recently published results from the largest prospective VCTE-based screening trial conducted to date [120]. This study invited 30,199 individuals aged ≥40 years from the general population to undergo screening at primary healthcare centres and screening units across nine European countries. Screening positivity was defined as an LSM ≥8.0 kPa or ALT ≥1.5 times the upper limit of normal, and screening-positive individuals were referred to hepatology. Of all participants, 1376 (4.6%) had an LSM ≥8.0 kPa, and 699 (2.3%) had an elevated ALT without an abnormal LSM. The prevalence of LSM ≥8.0 kPa was 14% in participants with type 2 diabetes and 3% in those without, and type 2 diabetes was the single strongest risk factor for LSM ≥10.0 kPa. However, similar to the Lindfors et al study [119], LSM was generally lower at the hepatology visit than at screening, and 715 of the 1491 re-tested participants (48%) had an LSM in the normal range below 8.0 kPa [120].

Taken together, mathematical projections and emerging real-world evidence suggest that, under the currently recommended screening algorithm, 30–50% of all individuals with type 2 diabetes require VCTE and approximately 15% are referred to hepatology. Critically, both modelled and observed data point to a high false-positive burden when using LSM ≥8.0 kPa to guide referral. Given the high prevalence of type 2 diabetes in high-income countries, these figures imply that large segments of the population could undergo unnecessary second-line testing and specialist evaluation, raising serious concerns about cost-effectiveness and feasibility [118].

## Evidence for and against fibrosis screening in type 2 diabetes

**Fig. 6:**
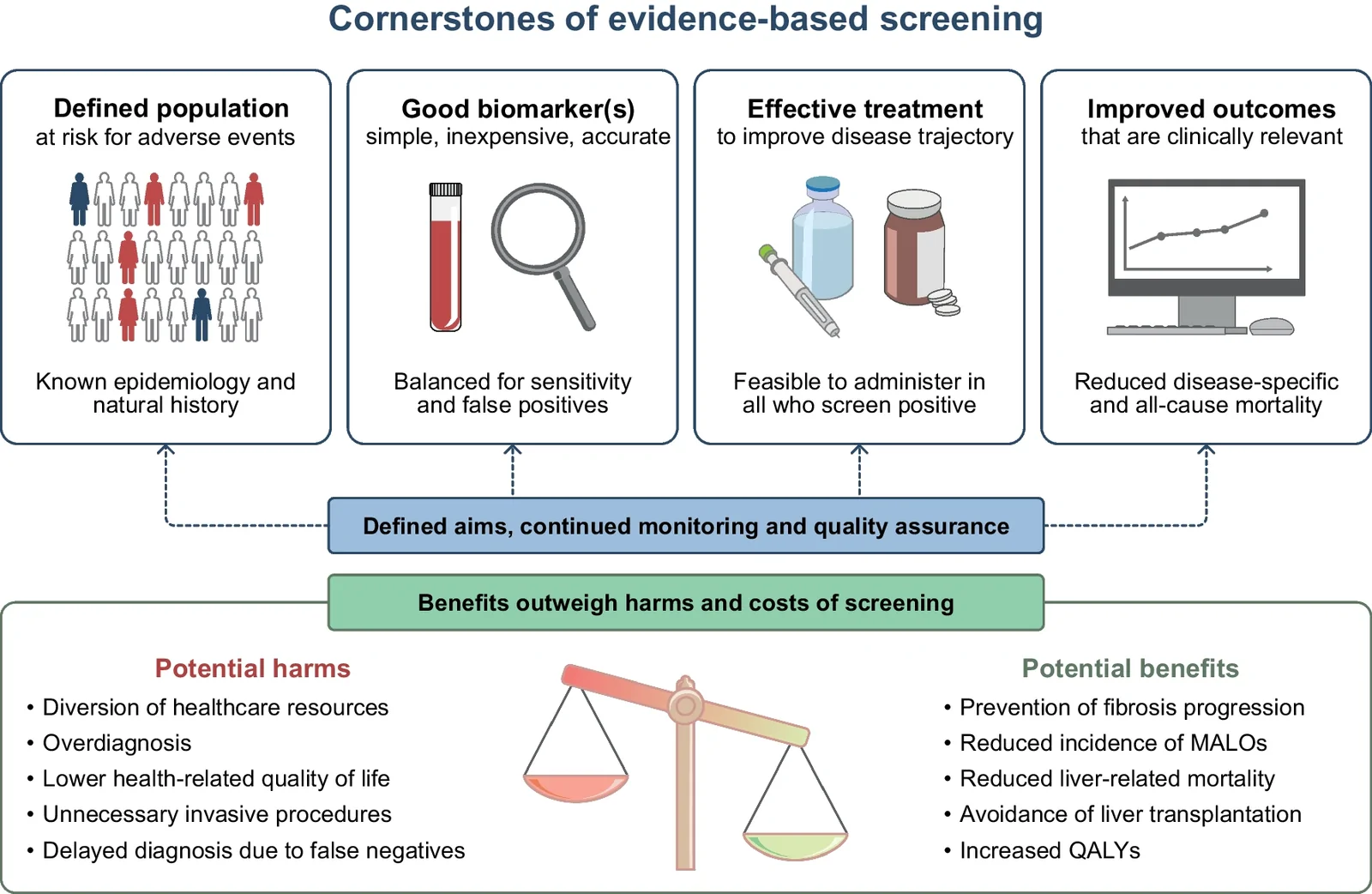
Cornerstones of a successful screening programme and potential harms and benefits. A recommendation for disease case finding should be based on evidence that key requirements are met. First, there should be a well-defined target population with a clinically significant increased risk of adverse events from the screened disease. To define such a population requires adequate knowledge of the epidemiology and natural history of the disease. Second, one or more biomarkers are required that are simple and inexpensive to measure, while being sufficiently accurate to detect the disease. A test and its cut-offs should be chosen to achieve a sufficiently high sensitivity while limiting the number of false positives requiring costly follow-up tests or specialist referrals. Third, an effective treatment should be available that can improve the disease trajectory and which is feasible to implement at scale for all individuals found to have the disease. Finally, case finding is predicated on the expectation that it will translate into clinically meaningful improvements in outcomes, ideally decreased risks of disease-specific and all-cause mortality. The potential benefits of screening should also outweigh the associated harms, as well as the costs of detection. This figure is available as part of a downloadable slideset

Figure 6 illustrates the core requirements for an effective screening programme. In 1968, Wilson and Jungner proposed ten criteria for the WHO that continue to underpin the evaluation of new screening initiatives today [121]. The WHO maintains that opportunistic case finding in routine clinical practice should be evaluated against the same rigorous scientific standards as national screening programmes, given its potential to generate both benefits and harms (Fig. 6) [122]. Table 3 presents the author’s assessment of these criteria as applied to AF screening in type 2 diabetes. While some requirements are clearly met, others are likely to fall short under scrutiny. Key concerns relate to whether the entire type 2 diabetes population represents the appropriate screening target, the performance of current NITs in a screening context, and the likelihood that screening will improve clinical outcomes. Importantly, no randomised controlled screening trials have demonstrated that AF case finding in type 2 diabetes, or in any population, improves outcomes or is cost-effective compared with no screening.

**Table 3:** Evaluation of fulfilment of the Wilson and Jungner criteria [121] for screening for AF in type 2 diabetes

No.	Criterion	Fulfilled?	Explanation
1	The condition should be an important health problem	No	AF itself carries significant risks of morbidity and mortality, but its prevalence in T2D is poorly characterised and may be markedly overestimated (Fig. 4). The 10 year risk of MALOs is <2% in unselected T2D populations, which is not clinically significant. This low disease burden is likely to limit the potential clinical impact of screening as well as the utility of NIT-based strategies (see criterion no. 5).
2	There should be an accepted treatment for patients with recognised disease	No^a^	Pharmacological treatments for at-risk MASH exist, but current agents (e.g. resmetirom, semaglutide) were approved on histological surrogate endpoints with confirmatory outcome trials ongoing, and they are licensed only for non-cirrhotic F2/F3 disease. Lifestyle intervention for weight loss and GLP-1 RA therapy are already indicated for many individuals with T2D. While identifying AF might plausibly intensify or prioritise such treatments, this added benefit is unquantified. Screening may therefore not meaningfully alter management in a significant proportion of cases, limiting the value of early detection and potentially worsening the harm-to-benefit ratio. Surveillance for HCC and oesophageal varices in those found to have AF could enable earlier treatment of liver-related complications. Critically, no randomised controlled trial has shown that delivering any of these interventions through a screening pathway improves prognosis.
3	Facilities for diagnosis and treatment should be available	Unclear	Healthcare systems may lack the resources required to accommodate the large volume of follow-up examinations that ensue when screening all T2D patients for AF using an algorithm with limited specificity (Fig. 5). Access to VCTE is often restricted to tertiary centres due to its high costs related to equipment acquisition and operator training. In addition, substantial added resources are required to deliver effective weight loss interventions and costly pharmaceuticals to individuals who screen positive, necessitating national policy-level planning before implementation of screening.
4	There should be a recognisable latent or early symptomatic phase	Yes	MASLD-related cirrhosis is preceded by a prolonged asymptomatic phase characterised by MASH and progressive fibrosis. This provides a theoretical window for early detection and intervention.
5	There should be a suitable test or examination	No	Fibrosis NITs have limited accuracy, with unacceptably high rates of false-positive results when used in low-prevalence populations (Figs 4 and 5) and limited sensitivity to detect AF. Second-line tests are only incrementally more accurate and remain non-diagnostic in pre-cirrhotic MASLD. The high false-positive rate precludes routine use of liver biopsy for confirmation in all individuals who test positive.
6	The test should be acceptable to the population	Unclear	Fibrosis NITs are generally safe, but indeterminate or discordant results may necessitate liver biopsy, introducing procedural risk and physical or psychological discomfort. Because current screening algorithms have low sensitivity, 30–40% of individuals with AF are classified as ‘low risk’, which is unacceptable for screening and may delay diagnosis and treatment due to false reassurance. In addition, unclear stopping rules for discordant or persistently elevated NIT results can lead to repeated cycles of imaging, which may further reduce acceptability to patients.
7	The natural history of the condition, including development from latent to declared disease, should be adequately understood	Yes	The natural history of MASLD and its progression from isolated steatosis to cirrhosis is adequately, albeit incompletely, understood.
8	There should be an agreed policy on whom to treat as patients	Unclear	Emerging pharmaceuticals are primarily indicated for individuals with MASH and F2/F3 fibrosis (at-risk MASH). Those with compensated cirrhosis may also benefit from weight loss and other therapies aimed at preventing cirrhosis-related complications. However, it remains unclear how NITs should be used to target treatment effectively. As clinical trials rely on histological endpoints, evidence-based NIT thresholds for treatment decisions remain lacking, and there is disagreement among experts on their use. Moreover, current screening guidelines target AF instead of at-risk MASH.
9	The cost of case finding (including diagnosis and treatment of patients diagnosed) should be economically balanced in relation to possible expenditure on medical care as a whole	No	No prospective randomised screening trials with long-term outcome data exist to support robust economic evaluations. Current cost-effectiveness analyses in T2D rely heavily on expert opinion and modelling assumptions that are likely to favour screening, including high AF prevalence, rapid disease progression and broad therapeutic benefit.
10	Case finding should be a continuous process and not a ‘once and for all’ project	Yes	As presently recommended by major clinical practice guidelines, AF case finding in T2D is meant to be a continuous process.

^a^Although accepted pharmacological treatments for at-risk MASH exist in principle, Wilson and Jungner emphasise that the treatment offered through screening should have demonstrated a positive effect on prognosis [121]

GLP-1 RA, glucagon-like peptide 1 receptor agonist; T2D, type 2 diabetes

### Is AF an important health problem in type 2 diabetes?

The importance of a condition as a target for screening rests on two complementary considerations [121]: whether undetected disease exposes affected individuals to substantial risks of premature death or disability, and whether the condition is prevalent enough to create a population-level burden that screening could reduce.

At the individual level, AF is clearly associated with clinically significant excess morbidity and mortality. Among 60-year-old men with stage F3 fibrosis, the 10 year risks of serious liver events and liver-related mortality are 6.2% and 3.1%, respectively, rising markedly to 45.4% and 23.9% with progression to cirrhosis (F4) (Fig. 1) [21]. Notably, non-liver-related mortality far outweighs liver-related mortality in non-cirrhotic MASLD (Fig. 1), which may be principally driven by cardiovascular disease and extrahepatic cancers [123]. Regarding the role of type 2 diabetes, prospective cohort studies have identified it as an independent predictor of more rapid fibrosis progression, as assessed by both histology and LSM-VCTE [92, 124]. Type 2 diabetes also confers a two- to fivefold increased rate of incident hepatic decompensation and HCC, with the greatest risk in individuals with suboptimal HbA_1c_ [125].

The population-level picture, however, is less compelling. As discussed above, AF prevalence in type 2 diabetes may be markedly lower than the frequently reported figures of 20–40% (Fig. 4). A lower than anticipated prevalence would not only diminish the potential public health benefit of screening but also degrade the performance of NITs (Fig. 5). Supporting evidence for a modest liver disease burden comes from a recent analysis of the Swedish National Diabetes Register, which captures approximately 90% of all individuals in Sweden with type 2 diabetes and is therefore highly representative of the broader type 2 diabetes population [93]. This study followed 406,770 participants with type 2 diabetes and 2,033,850 age-, sex- and county-matched control individuals without diabetes for more than 21 million person-years. The 10 year cumulative incidence of MALOs was 1.8% in those with type 2 diabetes compared with 0.8% in control participants [93]. Another study from the same group showed that, even in the metabolically highest-risk subgroup, that is, those with type 2 diabetes with five additional cardiometabolic risk factors, the 15 year incidence of MALOs remained below 3% [126]. By contrast, individuals newly diagnosed with type 2 diabetes with a similar cardiometabolic risk burden experience a cumulative incidence of major cardiovascular events approaching 30% within only 5 years [127].

These data suggest that liver-related clinical events are uncommon in type 2 diabetes, which limits the attainable public health benefits of AF screening while increasing the potential for harm (Fig. 6). They also call into question the validity of prior cost-effectiveness analyses that assume a particularly high burden of liver disease in this population (see below).

### Does diagnosing AF influence management in type 2 diabetes?

A core requirement of screening is a clearly defined aim, such as reducing mortality, disease incidence or disease severity [128]. Achieving this requires case finding that leads to scalable therapies capable of beneficially altering the disease course (Fig. 6). Classic examples include early intervention in diabetic retinopathy to prevent vision loss [129], curative treatment of early-stage breast cancer to reduce mortality [130] and treatment of precancerous lesions in cervical cancer to reduce incidence [131], with the last two having proven benefit in prospective screening trials.

In MASLD, the cornerstone of therapy is sustained weight loss of at least 5–10%, achieved through intensive lifestyle intervention including dietary modification and increased physical activity [6, 10, 11]. When steatosis is accompanied by inflammation, ballooning and significant fibrosis (i.e. at-risk MASH), these measures may be augmented with pharmacotherapy or bariatric surgery [7–9]. Importantly, however, similar interventions already constitute standard care in type 2 diabetes, with weight loss strongly recommended for its profound benefits for glycaemic management, potential for diabetes remission, and reduced risks of cardiovascular morbidity and mortality [93, 127, 132].

Once-weekly semaglutide 2.4 mg has recently been demonstrated to have efficacy for at-risk MASH over 72 weeks, meeting the primary endpoints of steatohepatitis resolution without worsening of fibrosis (63% with semaglutide vs 34% with placebo) and fibrosis reduction without worsening of steatohepatitis (37% vs 22%) [7]. Given its broad metabolic benefits, semaglutide is a valuable therapeutic addition, and a diagnosis of at-risk MASH may prompt or reinforce its use in eligible individuals with type 2 diabetes. However, semaglutide is already indicated in type 2 diabetes at doses up to 2.0 mg as adjunct therapy for glycaemic management or when type 2 diabetes is associated with cardiovascular or chronic kidney disease, and at a dose of 2.4 mg when BMI is ≥27 kg/m^2^ regardless of HbA_1c_ [133, 134]. Many individuals with type 2 diabetes may therefore already be receiving semaglutide at doses at or near that indicated for MASH, in which case a liver disease diagnosis made through screening may have limited implications for optimising this therapy.

Resmetirom, an oral thyroid hormone receptor beta-selective agonist, is the first liver-directed pharmacotherapy approved for at-risk MASH in the USA and Europe [8]. Compared with semaglutide, it resulted in more modest histological improvements over placebo at 52 weeks and, similarly, has yet to demonstrate benefits for MALOs. Unlike semaglutide, resmetirom is weight neutral and has not been shown to confer major metabolic benefits beyond an ~15% reduction in LDL-cholesterol [8]. Barriers to its rapid widespread adoption include the high cost (~$47,000/year in the USA), uncertain stopping rules, evolving reimbursement policies internationally and limited real-world experience [135]. Consequently, resmetirom cannot reasonably serve as the therapeutic foundation for an AF screening programme, and current evidence does not support AF screening based on its availability alone.

In summary, it remains unclear whether AF screening in type 2 diabetes meaningfully alters management in ways that could translate into improved outcomes at the population level. One downstream effect is that an AF diagnosis prompts surveillance for complications of advanced liver disease, namely gastro-oesophageal varices and HCC. The benefits of these surveillance programmes are themselves difficult to estimate given the limited trial evidence, especially for MASLD-related cirrhosis [136].

### Is AF screening in type 2 diabetes cost-effective?

In the absence of RCT evidence, cost-effectiveness analyses of AF screening must rely on complex modelling frameworks and assumptions that integrate data from diverse study designs across each step of the screening pathway. In addition, analysts must estimate the real-world utility and cost of each component, which can vary markedly across countries, healthcare systems and patient populations.

The economic modelling study by Noureddin et al [137] is frequently cited in support of the cost-effectiveness of AF screening [4, 10, 11, 57, 90, 138]. In a hypothetical cohort of 55-year-old individuals with type 2 diabetes, the authors concluded that a pathway based on ultrasound and measurement of aminotransferases followed by VCTE, with intensive lifestyle intervention for those with elevated LSM, was cost-effective, with an incremental cost-effectiveness ratio (ICER) of approximately $35,000 per quality-adjusted life-year (QALY) [137]. However, several modelling choices may limit the validity of this conclusion. The evaluated pathways differ from current recommendations, which prioritise FIB-4 as the initial test. The model assumed a MASLD prevalence in type 2 diabetes of 100%, with 64% having MASH with ≥F1 fibrosis and 16.1% having AF, figures that are likely to be overestimates (Fig. 4) [1, 2]. The benefit of intensive lifestyle intervention was modelled using stage-specific fibrosis regression rates of 77–100%, derived from the subgroup in the study by Vilar-Gomez et al who achieved ≥10% weight loss [6]—despite only one in ten participants reaching this threshold—and seemingly applied to all individuals receiving the intervention. The model further assumed sustained weight loss after 1 year of the intensive lifestyle intervention, with no parameters accounting for treatment uptake, adherence, dropout or weight regain. Finally, no intensive lifestyle intervention was assigned to the unscreened group, despite it being standard of care in people with type 2 diabetes. Even under these favourable assumptions, the simulated ICERs remained close to the willingness-to-pay threshold of $50,000 per QALY, with approximately 30% of probabilistic sensitivity analysis iterations failing to meet this threshold [137].

A more recent analysis by Younossi et al evaluated six guideline-recommended screening strategies and, in contrast to the study by Noureddin et al, modelled resmetirom ($47,400/year indefinitely) rather than intensive lifestyle intervention ($2865 for 1 year [137]) as the therapeutic intervention [139]. Despite the higher treatment cost, the authors concluded that AF screening in type 2 diabetes remains cost-effective, with ICERs of ~$27,000 per QALY [139]. The incremental QALY gain was ~0.51 per screened individual with type 2 diabetes, equivalent to 6 months of perfect health gained over 20 years. This estimate is ~50-fold larger than that reported by Noureddin et al (0.01) [137], reflecting the marked efficacy attributed to resmetirom therapy.

Several assumptions may have driven these favourable results. First, the baseline AF prevalence was set at 12.6%, which again is a possible overestimate [139]. More critically, the model projected an AF prevalence and cumulative mortality of ~47% and ~86%, respectively, without screening, compared with ~25% and ~80% with screening over a 20-year horizon. These rates of fibrosis progression and mortality are strikingly high and appear inconsistent with epidemiological data showing a 15 year incidence of MALOs in type 2 diabetes of <5% [93]. Second, resmetirom was modelled to confer a ~68% reduction in the probability of fibrosis progression and an ~82% increase in the probability of fibrosis regression annually, with both effects sustained across the entire time horizon [139]. These multipliers were derived from the 52 week MAESTRO-NASH trial, presumably based on overall fibrosis progression and regression rates in the resmetirom vs placebo arms [8], without accounting for factors such as waning of treatment effect or therapy discontinuation. Indeed, a deterministic sensitivity analysis identified the resmetirom treatment effect as the single most influential parameter [139]. However, the exact parameter ranges tested were not reported and no probabilistic sensitivity analysis was performed, leaving model robustness difficult to evaluate.

Taken together, existing cost-effectiveness analyses are undermined by assumptions that might systematically favour AF screening, including probable overestimation of fibrosis prevalence and progression rates, as well as highly optimistic treatment effects.

## Conclusions

An estimated 44 million people in Europe live with type 2 diabetes [140]. According to the data presented in this review, full implementation of currently recommended AF screening algorithms could result in approximately 13–22 million people (30–50%) undergoing VCTE and 7 million (15%) being referred to tertiary care, many of whom would need repeat testing. These figures stand in stark contrast to the less than 1 million individuals (1.8%) who are expected to develop MALOs over the next 10 years [93]. Given this scale, widespread adoption of AF screening requires robust evidence from clinical trials and economic modelling studies grounded in real-world data [128]. Several ongoing prospective screening trials are actively generating such evidence [115, 120], and large consortia such as LITMUS and NIMBLE continue efforts to identify NITs with greater accuracy and specificity to improve their utility in lower prevalence populations.

MASLD is undeniably an important comorbidity of type 2 diabetes. Hepatic insulin resistance is central to the metabolic syndrome and drives key cardiovascular risk factors, including dysglycaemia and atherogenic dyslipidaemia, and cardiovascular mortality is markedly increased in this population [141]. This association is clinically informative, as the presence of the metabolic syndrome can be taken as a strong indicator of underlying hepatic steatosis. It also highlights that the dominant health burden of MASLD lies outside the liver. Although liver-related mortality sees the greatest relative increase among cause-specific mortalities in MASLD, the vast majority of affected individuals are destined to die from CVD or extrahepatic malignancy rather than cirrhosis-related complications [123]. This reality is well recognised by diabetologists and primary care physicians and may clash with expert narratives that overemphasise the cirrhosis-related risk, potentially undermining their credibility and weakening adherence to future guidelines.

These considerations shift the focus towards a more constructive question: how can existing diagnostic and therapeutic tools best support the minority of people who will progress to clinically significant liver disease? The greatest gains are likely to come from strategies already known to be effective, including population-level policies that promote healthy lifestyles and, within healthcare systems, aggressive treatment of obesity. Indeed, adherence to current type 2 diabetes management guidelines alone should substantially reduce the burden of MASLD. It remains possible that a clearly defined subgroup of type 2 diabetes is at sufficiently high risk of AF to justify targeted case finding [108], but such a group is not yet well characterised. In the meantime, existing NITs may be most valuable not as triggers for referral, but as tools to target evidence-based therapies to those at highest risk of progressive liver disease, long before involvement by the hepatologist. Screening, by contrast, offers only potential benefit while inevitably introducing harm (Fig. 6) and, in the absence of stronger evidence, a risk-based approach within comprehensive cardiometabolic care is more consistent with clinical reality.

## Supplementary Information

Below is the link to the electronic supplementary material.

ESM (PDF 258 KB)

Slideset of figures (PPTX 1.05 MB)

## Abbreviations

AF: Advanced fibrosis
ALD: Alcohol-associated liver disease
ALT: Alanine aminotransferase
AST: Aspartate aminotransferase
CAP: Controlled attenuation parameter
FNI: Fibrotic NASH Index
HCC: Hepatocellular carcinoma
ICER: Incremental cost-effectiveness ratio
LITMUS: Liver Investigation: Testing Marker Utility in Steatohepatitis
LSM: Liver stiffness measurement
MALO: Major adverse liver-related outcome
MASH: Metabolic dysfunction-associated steatohepatitis
MASLD: Metabolic dysfunction-associated steatotic liver disease
MetALD: Metabolic dysfunction and alcohol-associated liver disease
MRE: Magnetic resonance elastography
NAFLD: Non-alcoholic fatty liver disease
NIMBLE: Non-Invasive Biomarkers for Metabolic Liver Disease
NIT: Non-invasive test
PDFF: Proton density fat fraction
QALY: Quality-adjusted life-year
VCTE: Vibration-controlled transient elastography
