# Supplementary material for: Screening and diagnosis of liver disease in diabetes: a guide and evidence-based assessment
Source: Diabetologia. 2026 Aug 6;69(11):3048–72. doi: 10.1007/s00125-026-06816-0 (PMC13627240; doi:10.1007/s00125-026-06816-0)
Supplement: Supplementary file 1 [file 125_2026_6816_MOESM1_ESM.pdf]

Screening and diagnosis of liver disease in diabetes: a guide and evidence-based assessment

Sami F. Qadri

Electronic supplementary material

Table of Contents

ESM Methods *2*

Bayesian approach for determining true AF prevalence from LSM-based estimates 2

ESM Tables *4*

ESM Figures *5*

References *6*

ESM Methods

Bayesian approach for determining true AF prevalence from LSM-based estimates

To illustrate the impact of imperfect NIT performance on LSM-based AF prevalence estimates, we applied the Bayesian framework described by Speybroeck *et al.* [1] to previously published data from prevalence studies in T2D populations. Specifically, we re-analyzed the studies summarized in Table 1 of [2], in which AF was defined by either VCTE (cut-off 9.6–9.7 kPa) or MRE (cut-off 3.60–3.63 kPa), extracting for each study the number of tested individuals (n) and the number of positive NIT results (x).

For each study, we modeled the observed number of positive NIT results as

$$x \sim \text{Binomial}(n,\ p) \quad (1)$$

$$p = \pi \times Se + (1 - \pi) \times (1 - Sp) \quad (2)$$

where p is the observed AF prevalence, π is the true AF prevalence, and Se and Sp are the sensitivity and specificity of the NIT at the relevant cut-off. This formulation directly relates the observed proportion of positive NIT results to the underlying true prevalence through the Rogan–Gladen estimator [3], with the unknowns π, Se, and Sp jointly estimated within a single probabilistic model.

A non-informative uniform prior over the interval [0, 1] was placed on π, expressing the absence of prior assumptions regarding the true AF prevalence. Informative Beta priors were placed on Se and Sp, parameterized to reflect plausible ranges for each NIT at the relevant cut-off. To inform these priors, we extracted Se and Sp estimates from individual participant data meta-analyses of VCTE and MRE diagnostic accuracy at the specified cut-offs (ESM Table 1) [4-7]. As multiple estimates were available, we selected those most favorable to each NIT, so that the priors, if anything, would overestimate rather than underestimate NIT performance. Hyperparameters of the Beta priors were derived using the epi.betabuster function in the R package epiR, which returns the Beta distribution corresponding to a specified mode and a defined probability of exceeding a lower bound. For MRE at 3.60–3.63 kPa, Se was assigned a mode of 0.83 with 95% probability of

exceeding 0.75 (Beta(69.42, 15.01)), and Sp a mode of 0.88 with 95% probability of exceeding 0.80 (Beta(63.66, 9.54)). For VCTE at 9.6–9.7 kPa, Se was assigned a mode of 0.77 with 95% probability of exceeding 0.70 (Beta(92.62, 28.37)), and Sp a mode of 0.81 with 95% probability of exceeding 0.75 (Beta(118.45, 28.55)). The resulting prior densities are shown in ESM Fig 1. The same priors were applied to all studies using a given NIT and threshold.

Posterior distributions were sampled using Hamiltonian Monte Carlo as implemented in Stan (version 2.32.2) via the R interface rstan (version 2.32.7). For each study, four independent chains were run for 100,000 iterations each, with the first half of each chain discarded as warm-up. Convergence was assessed by the Gelman–Rubin statistic ($\hat{R} < 1.01$ for all parameters), the effective sample size, and visual inspection of trace plots. For each study, we show the posterior mean and 95% credible interval (2.5th–97.5th posterior percentiles) of π.

All analyses were performed in R version 4.5.3.

ESM Tables

ESM Table 1. Diagnostic performance parameters for VCTE and MRE to detect AF, as reported by individual participant data meta-analyses.

**Test**	**Author, year**	***n***	**Cut-off for AF**	**Sensitivity (95% CI)**	**Specificity (95% CI)**	**Population (T2D/Mixed)**	**Ref.**
VCTE	Mózes *et al.*, 2022	5489	9.6 kPa	73 (71–76)	81 (79–81)	Mixed	[5]
	Pennisi *et al.*, 2023	1692	9.7 kPa	77 (74–80)	71 (68–74)	T2D	[6]
MRE	Hsu *et al.*, 2019	230	3.62 kPa	83 (73–92)	83 (78–89)	Mixed	[4]
	Liang *et al.*, 2023	266*	3.62 kPa	78 (66–88)	88 (82–92)	Mixed	[7]

All of the diagnostic accuracy studies cited here used liver biopsy as the reference standard.

*In the study by Liang *et al.*, the 3.62 kPa cut-off was investigated only in a subgroup of the entire cohort.

Abbreviations: AF, advanced fibrosis; CI, confidence interval; VCTE, vibration-controlled transient elastography; MRE, magnetic resonance elastography; T2D, type 2 diabetes.

ESM Figures

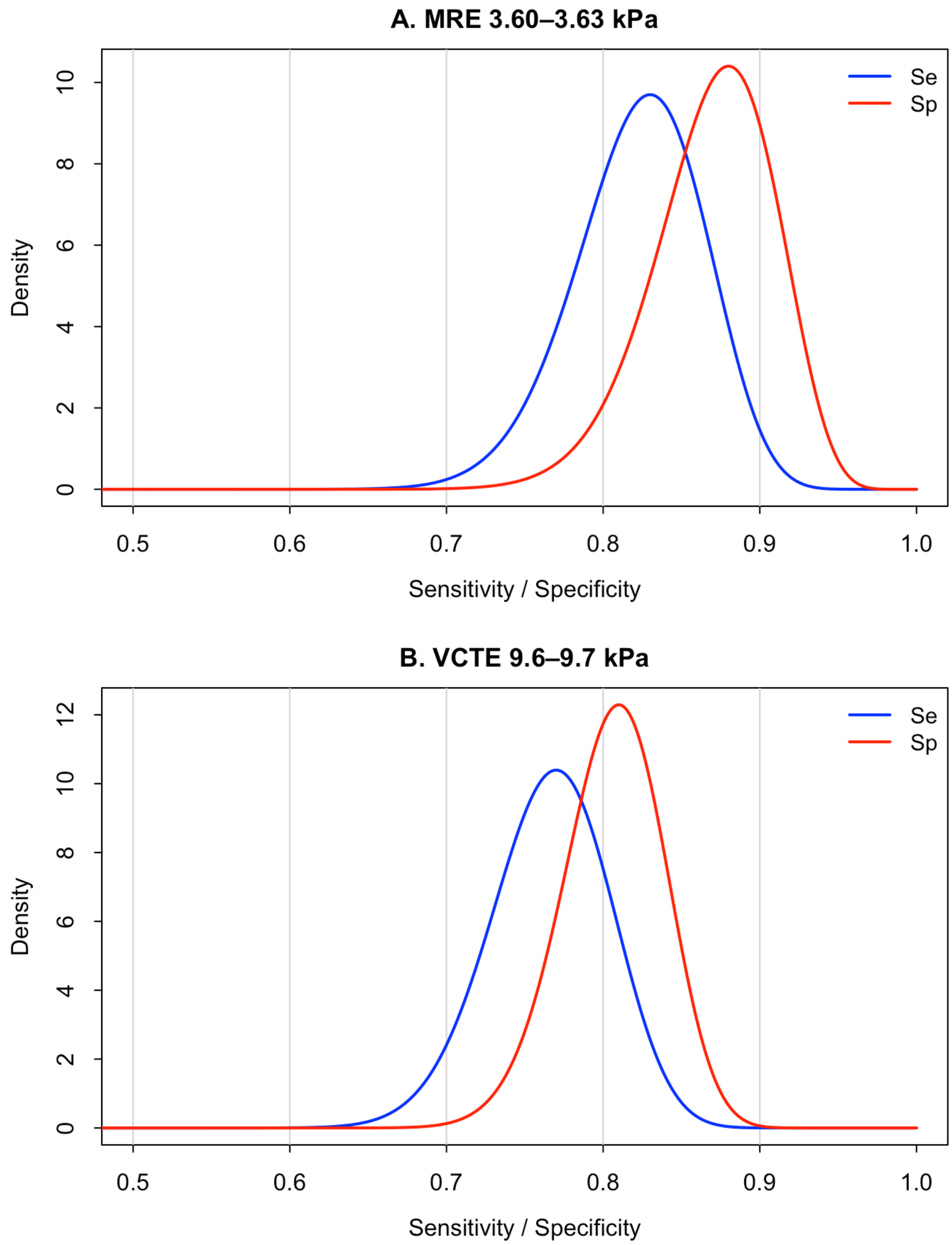

ESM Fig 1. Informed Beta prior distributions for the sensitivities and specificities of MRE and VCTE used in the Bayesian analysis. (**A**) For MRE: Se ~ Beta(69.42, 15.01); Sp ~ Beta(63.66, 9.54). (**B**) For VCTE: Se ~ Beta(92.62, 28.37); Sp ~ Beta(118.45, 28.55).

References

[1] Speybroeck N, Devleesschauwer B, Joseph L, Berkvens D (2013) Misclassification errors in prevalence estimation: Bayesian handling with care. Int J Public Health 58(5): 791–795. 10.1007/s00038-012-0439-9

[2] Qadri S, Yki-Jarvinen H (2024) Surveillance of the liver in type 2 diabetes: important but unfeasible? Diabetologia 67(6): 961–973. 10.1007/s00125-024-06087-7

[3] Rogan WJ, Gladen B (1978) Estimating prevalence from the results of a screening test. Am J Epidemiol 107(1): 71–76. 10.1093/oxfordjournals.aje.a112510

[4] Hsu C, Caussy C, Imajo K, et al. (2019) Magnetic Resonance vs Transient Elastography Analysis of Patients With Nonalcoholic Fatty Liver Disease: A Systematic Review and Pooled Analysis of Individual Participants. Clinical gastroenterology and hepatology : the official clinical practice journal of the American Gastroenterological Association 17(4): 630–637 e638. 10.1016/j.cgh.2018.05.059

[5] Mozes FE, Lee JA, Selvaraj EA, et al. (2022) Diagnostic accuracy of non-invasive tests for advanced fibrosis in patients with NAFLD: an individual patient data meta-analysis. Gut 71(5): 1006–1019. 10.1136/gutjnl-2021-324243

[6] Pennisi G, Enea M, Falco V, et al. (2023) Noninvasive assessment of liver disease severity in patients with nonalcoholic fatty liver disease (NAFLD) and type 2 diabetes. Hepatology 78(1): 195–211. 10.1097/HEP.0000000000000351

[7] Liang JX, Ampuero J, Niu H, et al. (2023) An individual patient data meta-analysis to determine cut-offs for and confounders of NAFLD-fibrosis staging with magnetic resonance elastography. J Hepatol 79(3): 592–604. 10.1016/j.jhep.2023.04.025
